# TRIM26 deficiency enhancing liver regeneration through macrophage polarization and β-catenin pathway activation

**DOI:** 10.1038/s41419-024-06798-0

**Published:** 2024-06-26

**Authors:** Tingting Li, Wei Zhong, Mengqi Li, Zile Shao, Gongye Zhang, Weiwei Wang, Zhixing Gao, Xuemei Tan, Ziyi Xu, Fanghong Luo, Gang Song

**Affiliations:** Cancer Research Center, School of Medicine, Xiamen University, Xiamen, China

**Keywords:** Hepatocyte growth factor, Growth factor signalling

## Abstract

Liver regeneration is a complex process involving the crosstalk between parenchymal and non-parenchymal cells, especially macrophages. However, the underlying mechanisms remain incompletely understood. Here, we identify the E3 ubiquitin ligase TRIM26 as a crucial regulator of liver regeneration. Following partial hepatectomy or acute liver injury induced by carbon tetrachloride, *Trim26* knockout mice exhibit enhanced hepatocyte proliferation compared to wild-type controls, while adeno-associated virus (AAV)-mediated overexpression of *Trim26* reverses the promotional effects. Mechanistically, *Trim26* deficiency promotes the recruitment of macrophages to the liver and their polarization towards pro-inflammatory M1 phenotype. These M1 macrophages secrete Wnts, including Wnt2, which subsequently stimulate hepatocyte proliferation through the activation of Wnt/β-catenin signaling. In hepatocytes, *Trim26* knockdown reduces the ubiquitination and degradation of β-catenin, thereby further enhancing Wnt/β-catenin signaling. Pharmacological inhibition of Wnt/β-catenin pathway by ICG-001 or depletion of macrophages by clodronate liposomes diminishes the pro-regenerative effects of *Trim26* deficiency. Moreover, bone marrow transplantation experiments provide evidence that *Trim26* knockout in myeloid cells alone can also promote liver regeneration, highlighting the critical role of macrophage *Trim26* in this process. Taken together, our study uncovers TRIM26 as a negative regulator of liver regeneration by modulating macrophage polarization and Wnt/β-catenin signaling in hepatocytes, providing a potential therapeutic target for promoting liver regeneration in clinical settings.

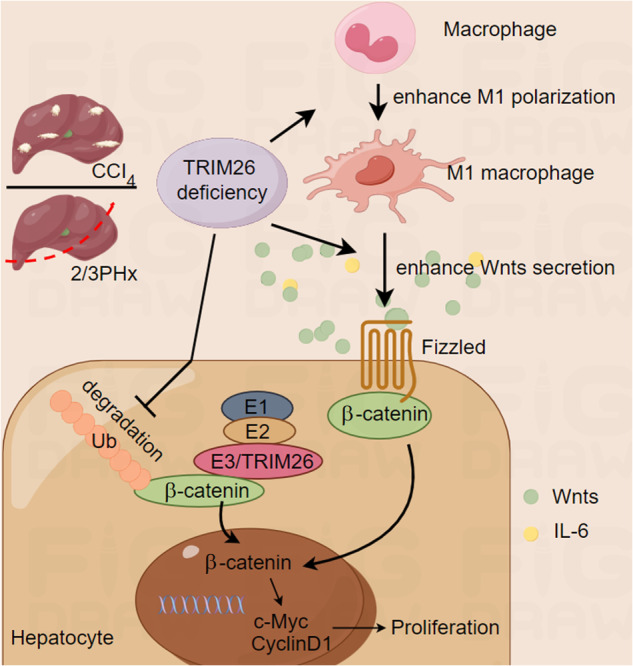

## Introduction

The liver is a unique organ capable of self-renewal of mature cells and maintaining liver homeostasis after surgical resection or toxin-induced injury. In mouse experiments, the liver can be restored to its original size within approximately 7–14 days after partial hepatectomy (PHx) [1]. Following resection, hepatocytes exhibit a proliferative response originating from the portal vein region and extends towards the central periphery [2, 3]. This regenerative process is complex and involves the crosstalk of multiple cell types, including parenchymal and non-parenchymal cells [4]. Recent studies have highlighted the significant role of non-parenchymal cells, particularly innate immune cells, in liver regeneration [5]. However, the mechanisms regulating liver regeneration, especially the crosstalk between hepatocytes and immune cells, remain completely understood.

In the context of hepatectomy and liver injury, inflammation has been found to play a major role in the regulation of hepatic regeneration. It serves a protective role by facilitating the removal of cellular waste and stimulating regenerative processes [6]. However, excessive inflammation can have detrimental effects on various liver diseases by exacerbating liver damage [7]. Therefore, understanding the intricate balance of inflammation is crucial for effective therapeutic interventions.

Macrophages, as the predominant immune cells in the liver, have a crucial function in maintaining hepatic homeostasis and understanding the mechanisms involved in liver diseases [8]. The equilibrium between M1 and M2 polarization is pivotal in hepatic inflammation and repair processes, making strategies targeting macrophage polarization potentially effective for alleviating acute liver injury [3]. For instance, pro-inflammatory M1 macrophages have been found to induce harm to neighboring tissues and impede the regeneration of surrounding cells by secreting pro-inflammatory cytokines and chemokines that activate intracellular cell death mechanisms [9]. On the other hand, they can also produce pro-inflammatory cytokines, including IL-6, TNF-α, and IL-1β, that are critical for supporting liver regeneration [10, 11]. M2 macrophages secrete anti-inflammatory cytokines, which can reduce inflammation and promote tissue repair [3]. To gain a comprehensive understanding of liver diseases and develop effective therapeutic interventions, it is essential to unravel the underlying mechanisms of macrophage polarization and their interactions with hepatic cells. This knowledge can help identify novel targets and develop targeted therapies for liver injury and regeneration.

The Wnt/β-catenin pathway is increasingly recognized as a significant regulator of metabolic zonation, homeostatic renewal, and regeneration in the liver in response to injury [12]. Labeling experiments have demonstrated that approximately 30% of hepatocytes originate from the pericentral AXIN2 population after one year of liver homeostasis [13]. Deletion of Wnt2 and Wnt9b or Wntless in liver endothelial cells, or β-catenin or LRP 5-6 in liver cells, resulted in the absence of pericentral genes and alteration of periportal gene expression [14]. Ubiquitin is the main mechanism for regulating the quantity of β-catenin in Wnt signaling. In the absence of Wnt protein, the phosphorylation of β-catenin by CK1 and GSK3 leads to its ubiquitination through the binding of β-Trcp, an E3 ligase, and subsequent degradation by the 26S proteasome system [15, 16].

In our recent study, we investigated the role of the E3 ubiquitin ligase Tripartite motif-containing protein 26 (TRIM26) in promoting the ubiquitination of β-catenin, ultimately inhibiting the progression of hepatocellular carcinoma [17]. TRIM26, as a member of the TRIM family, shares similar structural features with other TRIM proteins, including a ring domain, one or two B-box domains, N-terminal coiled coil domains, and a variable C-terminal domain. Notably, TRIM26 is closely associated with innate immune. Upon viral infection, the TRIM26 protein undergoes nuclear translocation and interacts with the K48 residue of the IRF3 protein, thereby facilitating polyubiquitination and subsequent degradation [18]. TRIM26 expression is commonly reduced in various cancers and promote proliferation, while its overexpression has been shown to impede the proliferation of thyroid cancer cells, bladder cancer, hepatocellular carcinoma, and others [19–21]. The function of TRIM26 in the context of acute liver injury and liver regeneration remains unclear.

In this study, TRIM26 expression significantly decreased in proliferation on the third day after PHx. Additionally, the absence of *Trim26* resulted in enhanced liver regeneration in both liver resection and liver injury model, when compared to WT mice. We also noticed an increase in M1 macrophage infiltration in the liver after liver resection or injury when *Trim26* deficiency was present. Mechanistically, our study showed that the absence of *Trim26* leads to an elevation in the population of M1 macrophages and hinders the polarization of M2 macrophages. These M1 macrophages, characterized by *Trim26* deficiency, facilitate hepatocyte proliferation by secreting Wnts. In hepatocytes, the lack of *Trim26* enhances hepatocyte proliferation. This effect is attributed to the reduction in the ubiquitination of β-catenin, subsequently promoting the proliferation of liver cells. Based on our findings, it is postulated that the absence or inhibition of *Trim26* may serve as a theoretical foundation for the occurrence of liver injury or the necessity of liver transplantation.

## Materials and methods

### Animal and animal model

All animal experiments were reviewed and approved by Animal Care and Use Committees of Xiamen University. The *Trim26*^*−/−*^ mice were generated by Cyagen Biosciences Inc. *Trim26*^−/−^ mice and wild-type (WT) littermate controls, aged 6-10 weeks, were utilized in the study. All experimental mice were maintained in specific pathogen-free conditions and acclimated on 12 h dark-light cycles with temperature (22 ± 2 °C) and humidity (55 ± 5%).

For the CCl_4_ induced acute liver injury model, mice were administered an intraperitoneal injection of a 20% solution of CCl_4_ and sterile olive oil (Sigma-Aldrich, St. Louis, MO) at a dosage of 4 μL/g of animal weight. Mice were euthanized at different time intervals (1 day, 2 days, 3 days, 5 days, 7 days) following the administration of CCl_4_. ICG-001 (Selleck, Houston, USA) was injected intraperitoneally into mice at a dose of 20 mg/kg, once every 2 days, for three times in total. Subsequently, CCl_4_-induced liver injury was performed in mice. For macrophage depletion, the experimental group received Clodronate Liposomes (Yeasen, Shanghai, China), while the control group received liposomes containing only phosphate-buffered saline (PBS, Yeasen, Shanghai, China). The day before CCl_4_ treatment, each animal received an intraperitoneal injection of 0.2 mL of Clodronate Liposomes or PBS liposomes.

Two-third partial hepatectomy (PHx) was performed as described by Mitchell and Willenbring [22]. In brief, mice were sedated with inhaled Isoflurane and underwent a mid-ventral laparotomy procedure, which involved making an incision in the abdominal wall to expose the internal organs, followed by the resection of the left and median hepatic lobes. Sham-laparotomy-operated mice were utilized as the control group. The liver-to-body weight ratios were determined by calculating the weight of the remnant liver relative to the body weight. Adeno-associated virus serotype 8 (AAV8) under the control of the thyroid binding globulin promoter, which specifically infects hepatocytes, was used to overexpress *Trim26* in the hepatocytes of mouse livers. Adeno-associated viruses, approximately 10^11^ viral particles per dose, were delivered into mice via tail vein injection. Six weeks later, PHx was performed.

### Cell culture, plasmids and transient transfection

The L02 cells, AML12 cells, THP-1 cells, and Raw264.7 cells were cryopreserved in the laboratory. The L02 cells were cultured in DMEM (Gibco, USA) supplemented with 10% FBS (Gibco, USA). THP-1 cells and Raw264.7 cells were cultured in RPMI-1640 (Gibco, USA) supplemented with 10% FBS. The AML12 cells were maintained in DMEM/F12 (1:1) (Gibco, USA) with 10% FBS, 1% ITS, and 40 ng/mL dexamethasone. The cells were maintained in a controlled environment at 37 °C with 5% CO_2_. Additionally, the cells were regularly screened for mycoplasma contamination.

The shRNA primers were designed according to the pLV-RNAi system, utilizing the following sequences: shRNA#1 GATGGATGACGACTGGGAA and shRNA#2 GCTGCTGAGAGACTTGGAATA. The lentivirus vector pLV- *Trim26* -Puromycin or pLV-control-Puromycin is packaged into lentivirus together with plasmids pMDLg/pRRE, pVSV-G and pRSC-Rev. The lentivirus that had been packaged was subsequently transfected into 293T cells. The collected lentivirus infected L02 or Raw264.7 cells. Stable transfected cells were screened using puromycin following established protocols. Transient transfection was performed with Lipofectamine 8000 (Beyotime, C0533) for L02 cells. Cells were harvested 24 h after transfection.

### Western blot analysis

Whole cell lysates were extracted by RIRA lysis buffer (Beyotime Biotechnology, Beijing, China) with the addition of a cocktail of protease inhibitors (ROCHE, Basel, Switzerland). Western blot analyses were performed with anti-CyclinD1 (Cell Signaling Tech, 55506), anti-CyclinA2 (Cell Signaling Tech, 81754), anti-CyclinB1 (Cell Signaling Tech, 12231), anti-CyclinE1 (Cell Signaling Tech, 20808), anti-β-catenin (Santa Cruz Biotechnology, sc-133239), anti-c-Myc (Proteintech, 10828-1), anti-GPADH (Proteintech, 60004-1), anti-TRIM26 (Proteintech, 27013-1), anti-β-actin (Sigma-Aldrich, A3854) antibodies. HRP-conjugated anti-rabbit (Sigma-Aldrich, A0545) or mouse (Sigma-Aldrich, A05795) were used as secondary antibodies. Signal was detected using enhanced chemiluminescence (Thermo Fisher Scientific, USA) and the resulting protein bands were observed with the ChemiDocTM MP Imaging System (Bio-Rad Laboratories, California, USA).

### Isolation of primary hepatocytes, cell culture and treatments

Mouse primary hepatocytes were isolated from 6 to 8-week-old mice using collagenase Type II (Sigma-Aldrich, St. Louis, MO) in situ perfusion. The isolation procedure was performed on *Trim26*^−/−^mice or WT mice using a modified two-step method [23]. Mouse primary hepatocytes were cultured with DMEM with 10% FBS, Penicillin (100 U/mL), Streptomycin (100 μg/mL) for 24 h. IL-6 (10 ng/mL, Biolegend, San Diego, USA) was administered to hepatocytes for 24 h. ICG-001(10 µM) was administered to L02 for 24 h. HGF (50 ng/mL, Biolegend, San Diego, USA) was used for hepatocytes for 24 h. IWP-2(5 µM, Selleck, Houston, USA) was was administered to Raw264.7 for 24 h.

### Mouse primary bone marrow isolation and Bone Marrow Transplantation

C57BL/6 male mice, aged 6–8 weeks, were subjected to anesthesia and euthanized in order to collect bone marrow (BM). BM cells were extracted from the tibias and femurs by flushing with culture medium.

C57BL/6 male mice were housed in a specific-pathogen-free environment. Mice were exposed to total body irradiation using G-radiation from a ^60^Co irradiator at a dosage of 8 Gy (Rad source-2000pro, USA). Subsequently, mice were intravenously injected with 5 × 10^6^ BM cells through the tail vein. Eight weeks later, mice were used in experiments.

### Histology and immunochemistry

Liver tissue samples were collected and preserved in 4% formalin, followed by fixation and embedding processes. The 5 µm sections were stained with hematoxylin and eosin in order to conduct a morphological examination. Additionally, anti-Ki67 (Proteintech, 28074-1) were utilized for immunostaining. Five randomly selected microscope fields were analyzed using Image-Pro Plus software (National Institutes of Health, Bethesda, MD, USA).

### qPCR

Total messenger RNA (mRNA) was extracted from mouse liver tissues, primary hepatocytes, L02 cells, and AML12 cells using Trizol reagent (Invitrogen, Carlsbad, CA), following the manufacturer’s instructions. After conducting reverse transcription with the ReverTra Ace® qPCR RT Kit (TOYOBO Life Science, Shanghai, China), quantitative real-time PCR was carried out using the SYBR Green PCR Master Mix (Invitrogen, Carlsbad, CA). GAPDH was used as a reference gene. The primers utilized for gene amplification are presented in Supplementary Table S1.

### Biochemical detection

Serum levels of alanine aminotransferase (ALT) and aspartate aminotransferase (AST) were measured using commercially available kits (BioSino Bio-Technology and Science Inc, Beijing, China) and an automated biochemistry analyzer (Rayto Chemray240, Shenzhen, China).

### Flow cytometry analysis

Single-cell suspensions were incubated for 30 min with the indicated antibodies, including anti-F4/80-APC, anti-CD11b-FTTC, anti- CD45-PE-CF594, anti-CD86-PE, anti-IA/IE-Percp, anti-Ly6C-PE-Cy7, anti- Gr-1-APC-CY7, anti- CD4-BV421, anti- Ly6G-BV605, anti- CD8-BV650. (BD Biosciences, San Jose, CA). After a 15-minute incubation period at room temperature in the dark, the cells were subjected to three washes with PBS. FACS was performed on Fortessa X-20 (BD Biosciences, San Jose, CA) and analyzed with FlowJo V10.

### Ubiquitination assay

Cells were transfected with HA-β-catenin and Flag-Ub plasmids for the purpose of conducting an in vivo ubiquitination assay, following the established method as described [24]. In brief, cells that received the specified treatment were subjected to a 6-hour incubation with MG132 (Invitrogen, Carlsbad, CA) and subsequently lysed using a denaturing lysis buffer. The samples were subjected to immunoprecipitation with an HA-tag antibody.

### Statistical analysis

Data are presented as means ± SD. To conduct a comparison of values obtained from three or more groups, the statistical method of one-factor analysis of variance (ANOVA) was employed. To compare values obtained from 2 groups, the Student *t* test was performed. All in vitro experiments were repeated at least three times. Statistical significance was taken at the *P* < 0.05 level.

## Results

### *Trim26* deficiency promotes liver regeneration after liver injury

To determine the biological role of TRIM26 in hepatic cell proliferation following injury, we first examined the expression of TRIM26 in mice 2/3 PHx model. In the process of liver regeneration, hepatic TRIM26 levels showed a dynamic alteration in response to injury. They reduced from day 2 to day 5 and bottomed out at day 3 after PHx in the livers of mice (Supplementary Fig. S1A). These findings suggest a potential association between TRIM26 and liver regeneration following hepatectomy.

**Fig. 1:**
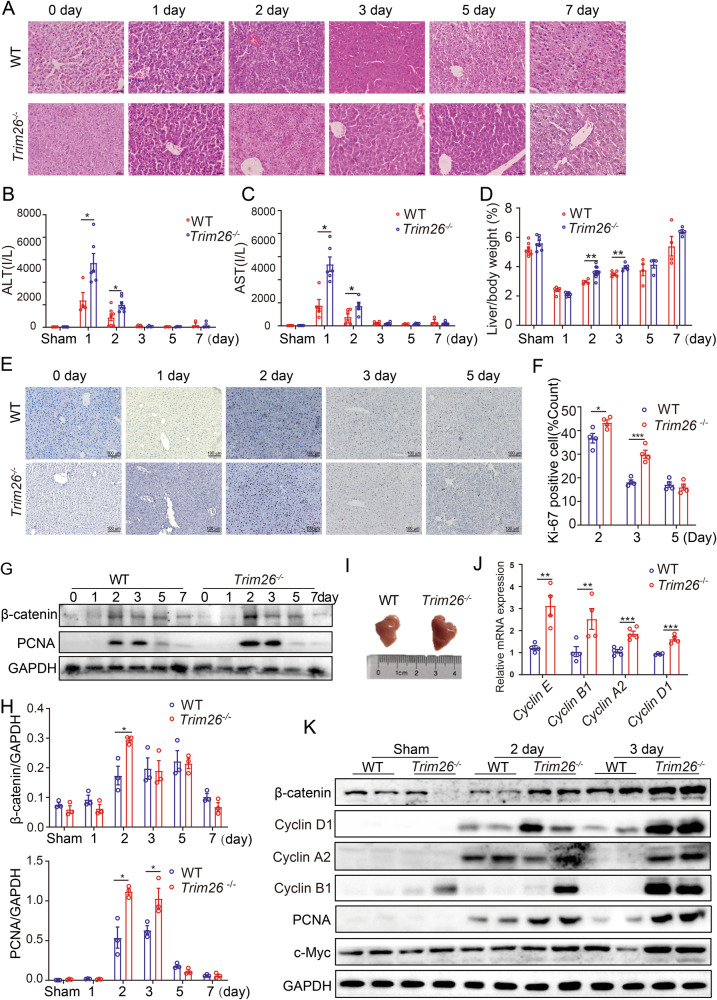
*Trim26* deficiency accelerates liver regeneration after hepatectomy. *Trim26*^−/−^ and WT mice were subjected to partial hepatectomy at the indicated time points. **A** Representative images of H&E staining. (*n* = 3, scale bar, 50 µm). **B**, **C** The serum levels of AST and ALT from WT and *Trim26*^−/−^ mice were detected. *n* = 3–7/group. **D** Liver-to-body weight ratios after hepatectomy. *n* = 4–7/group. **E**, **F** Representative images of Ki67 staining and quantification. (*n* = 4, scale bar, 100 µm). **G**, **H** Protein expression of hepatocyte proliferation such as PCNA and β-catenin. (*n* = 3). **I** The photograph of residual liver. **J** mRNA levels of cell cycle-related genes. *n* = 4-5/group. **K** Protein expression of cell cycle protein and β-catenin signaling. (*p < 0.05, **p < 0.01, ***p < 0.001).

We next performed PHx to induce liver regeneration in both WT and *Trim26*^−/−^ mice. *Trim26* deficiency showed more severe liver damage, as evidenced by a significant elevation of plasma levels of ALT and AST, augmented of lobular necrosis and inflammation (Fig. 1A–C, Supplementary Fig. S1D), and elevated kidney damage compared to the WT group (Supplementary Fig. S1D). In terms of hepatocyte proliferation, which plays a crucial role in the early stages of liver recovery during regeneration, we found that *Trim26*^*−/−*^ mice exhibited significantly higher liver-to-body weight ratio following hepatectomy, as shown in Fig. 1D. Remarkably, on the second day after hepatectomy, *Trim26* knockout mice exhibited an increase in Ki67-positive cells in comparison to the WT group (Fig. 1E, F). Moreover, the expression of proliferating cell nuclear antigen (PCNA) was found to be elevated at 2 days after PHx, and continued to increase until 3 days post PHx in comparison to WT controls (Fig. 1G, H). Given that activation of cell cycle plays a central role in stimulating robust hepatocyte proliferation during hepatic regeneration, we measured expression levels of the checkpoint components involved in controlling cell cycle progression. As depicted in Fig. 1J, K, the absence of *Trim26* resulted in a significant upregulation of cell cycle proteins, including Cyclin D1, Cyclin A2 and Cyclin E, after hepatectomy. This observation indicates an augmented progression from the quiescent G0 phase to the G1 phase.

Wnt/β-catenin signaling is an important driver of liver regeneration after hepatectomy. β-catenin protein rapidly increase and translocate to the nucleus in order to promote hepatocyte proliferation through the expression of target genes [25], such as cell-cycle regulator Cyclin D1 [26]. Notably, β-catenin protein was potently increased at 2 day after hepatectomy compared with WT (Fig. 1G), consistent with β-catenin signal downstream proteins (Fig. 1J). Taken together, these findings suggest that TRIM26 is reduced after partial hepatectomy and *Trim26* deficiency impairs liver damage and promotes liver regeneration.

**Fig. 2:**
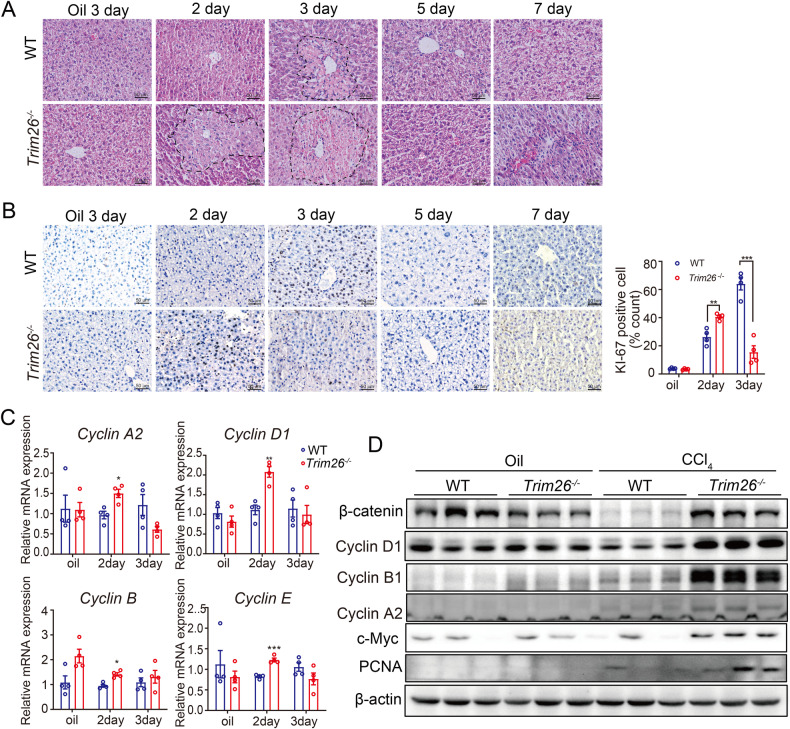
*Trim26* deficiency promotes hepatocyte proliferation in mice injected with CCl_4_. **A** Representative H&E staining was performed at the indicated time points. **B** Representative Ki67 staining and its statistical quantification. **C**, **D** mRNA levels and protein expression of cell cycle proteins and β-catenin signaling were examined during the process of liver recovery. *n* = 4/group, scale bar, 50 µm. (*p < 0.05, **p < 0.01, ***p < 0.001).

To further investigate the effect of *Trim26* on liver regeneration after liver injury, we performed another murine model of liver regeneration induced by intraperitoneal injection of CCl_4_. Compared to the WT group, *Trim26*-deficient mice showed more severe liver lobular injury, and increased lobular inflammation after CCl_4_ injection, as evidenced by H&E staining (Fig. 2A). Ki67-positive cells increased in the CCl_4_-induced acute liver injury model in *Trim26* deficient mice, suggesting a facilitated liver recovery (Fig. 2B). *Trim26* deficiency caused promotion of cell cycle genes after CCl_4_ injection, such as Cyclin D1, Cyclin A2 and Cyclin B1, suggesting an increased G1 to S phase transition (Fig. 2C, D). Taken together, these data demonstrate that the loss of *Trim26* causes the acceleration of liver regeneration and the rapid recovery of liver mass after CCl_4_ treatment.

### AAV-mediated overexpression of TRIM26 impedes liver regeneration after partial hepatectomy

**Fig. 3:**
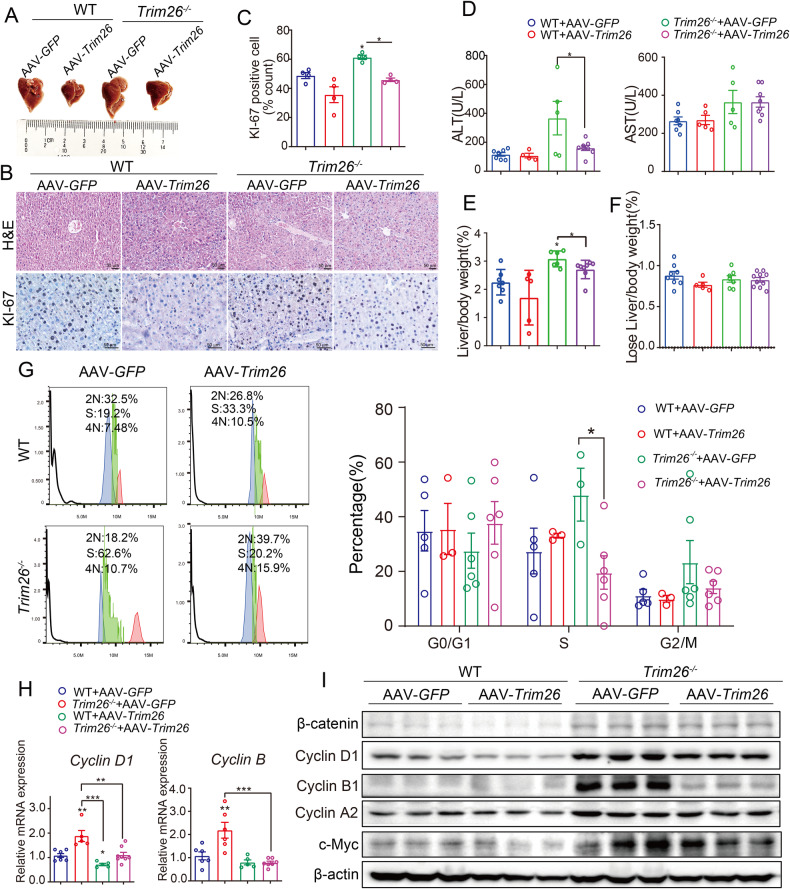
AAV-mediated overexpression of TRIM26 impedes liver regeneration in mice after partial hepatectomy. WT and *Trim26*^−/−^ mice were treated with adeno-associated virus (AAV) vectors encoding either TRIM26 or green fluorescent protein (GFP) via tail vein injection for 6 weeks. Subsequently, hepatectomy was performed. **A** The photograph of residual liver. **B**, **C** Representative H&E staining, Ki67 staining, and quantification. Scale bar, 50 µm. **D** Representative of plasma ALT and AST levels. **E** The liver/body weight ratio was determined at the indicated time points after PHx. **F** The detection of lose liver weight in liver sections. **G** Primary hepatocytes were isolated from *Trim26*^−/−^ and WT mice after partial hepatectomy, followed by flow cytometry analysis to measure the cell cycle. **H**, **I** mRNA levels and protein expression of cell cycle proteins and β-catenin signaling were decreased in AAV-TRIM26-treated mice after partial hepatectomy. *n* = 4–8/group. (*p < 0.05, **p < 0.01, ***p < 0.001).

To investigate the gain-of-function effects of TRIM26 on liver regeneration, we next utilized an adenoassociated virus (AAV) to mediate overexpression of TRIM26 in a mouse model of PHx. Six weeks after AAV-TRIM26 or AAV-GFP was injected into the tail vein, mice underwent PHx, and sacrificed at 2 days after hepatectomy (Fig. 3A). Compared to *Trim26*^−/−^ mice, the overexpression of *Trim26* resulted in a mild liver injury after hepatectomy, as evidenced by reduced lobular inflammation and necrosis, along with a potent reduction in plasma ALT levels (Fig. 3B, D). Moreover, the overexpression of TRIM26 inhibited hepatocyte proliferation, as demonstrated by decreased liver to body weight and diminished Ki67 staining (Fig. 3B, C, E). These findings suggest that hepatic overexpression of TRIM26 leads to attenuated recovery of liver function after hepatectomy.

Hepatocyte proliferation begins within 24 h and peaks at about 48 h after acute liver injury or hepatic resection [27]. As shown in Fig. 3G, overexpression of TRIM26 resulted in a decrease in G1 to S phase transition, leading to the inhibition of proliferation. Notably, qPCR and Western blot analysis showed that TRIM26 overexpression resulted in a marked reduction in cell cycle genes, such as *Cyclin D1, Cyclin E1*, and *Cyclin A*, which is consistent with the flow cytometry result (Fig. 3H, I). These results demonstrate that TRIM26 indeed inhibits liver regeneration in mice.

### The absence of TRIM26 increases the recruitment of macrophages to the liver and promotes M1 polarization

**Fig. 4:**
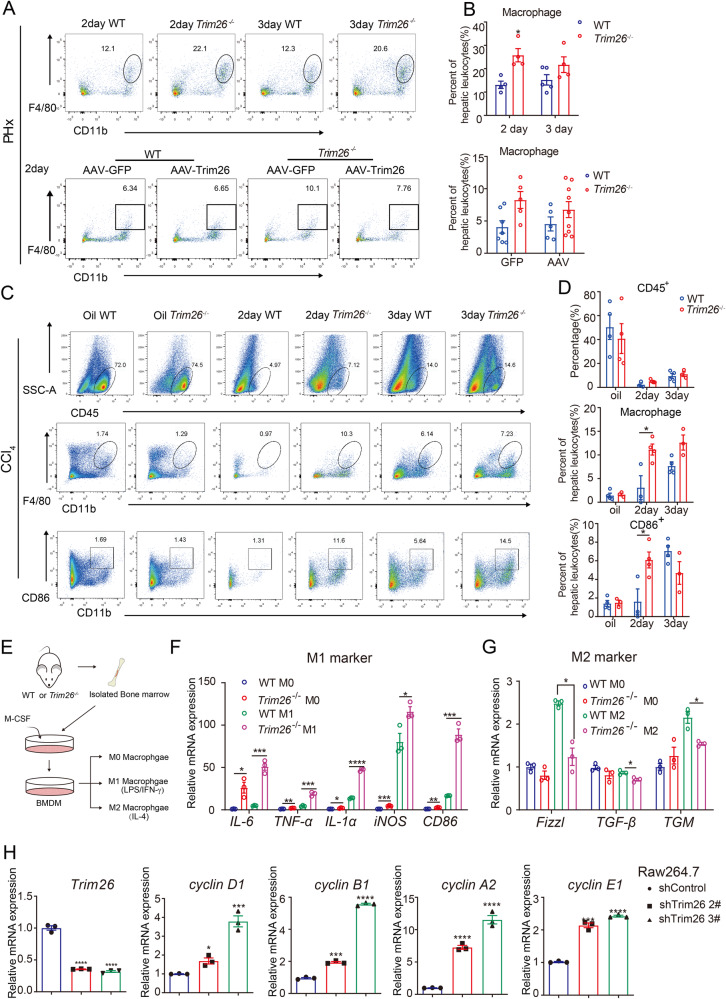
*Trim26* deficiency promotes M1 polarization and impedes M2 polarization in macrophages. **A**, **B** Representative macrophages (F4/80^+^, CD11b^+^) were harvested from *Trim26*^−/−^ and WT and AAV-TRIM26 mice 2 day after partial hepatectomy and the statistical quantification. **C**, **D** Representative macrophage (F4/80^+^, CD11b^+^) and (CD11b^+^, CD86^+^) profiles of CD45^+^ liver nonparenchymal cells harvested from *Trim26*^−/−^ and WT mice were gated and tested on the second day after CCl_4_ treatment and the statistical quantification. **E** Schematic overview of bone marrow-derived macrophages (BMDM) induced polarization. BMDM were stimulated with a concentration of IL-4 (20 ng/mL) or LPS (100 ng/mL) and IFN-γ (20 ng/mL). **F**, **G** The expression of M1 macrophage markers, including *IL-6, TNF-α, IL-β, iNOS*, and *CD86*, as well as M2 macrophage markers (*Fizzl, TGF-β*, and *TGM)*, was assessed using qPCR analysis. **H** RAW264.7 cells were transfected with control shRNA or shTrim26, and the expression of TRIM26 and cell cycle proteins was assessed by western blot. *n* = 3–9/group. (*p < 0.05, **p < 0.01, ***p < 0.001, ****p < 0.0001).

Next, we investigate the underlying mechanisms of the acceleration of liver regeneration phenotypes in *Trim26*^−/−^ mice. The microenvironment of liver plays an important role in the initiation of liver regeneration [28]. The population of immune cells in the liver after liver resection was analyzed by Flow cytometry. *Trim26* knockout in the sham group showed no significant difference compared to the WT group (Supplementary Fig. S2A). However, increased infiltration of macrophages (CD11b^+^F4/80^+^), MDSCs (CD11b^+^Gr-1^+^) and G-MDSCs (CD11b^+^LY6G^+^) was observed 2 days after surgery (Fig. 4A, B, Supplementary Fig. S2B). The number of white blood cells sharply decreased and then slowly increased following acute liver injury. We observed a higher number of hepatic white blood cells, macrophages, especially M1 macrophages in *Trim26*^*−/−*^ mice (Fig. 4C, D). In other words, *Trim26* deficiency resulted in increased infiltration of macrophages, especially M1 macrophages, in the liver. Treatment with clodronate liposomes to deplete macrophages led to a notable reduction in the quantity of macrophages in the liver and blood for both the WT group and the *Trim26* deficient group compared to the PBS liposomes (Supplementary Fig. S3A–D). As shown in Supplementary Fig. S3E–H, the depletion of macrophages reduced liver lobular inflammation and necrosis, as indicated by HE staining. The downregulation of liver cell proliferation was confirmed by Ki67 staining. The clearance of macrophages significantly reduced the β-catenin signal and cell cycle proteins, as demonstrated by qPCR and Western blot analysis.

Moreover, by detecting the RNA levels of M1 markers (*IL-6*, *CD86*, inducible nitric oxide synthase, *TNF-α*, *IL-1α*) and M2 markers (*Fizzl*, *TGM*, *TGF*-*β*) in induced isolated BMDM, we further confirmed the increased number of M1 macrophages and reduced M2 macrophages in *Trim26*^−/−^ mice (Fig. 4E–G). Deletion of *Trim26* in BMDM led to enhanced M1 polarization and reduced M2 polarization (Fig. 4E–G). To further clarify the underlying mechanisms driving the observed increase in macrophages, we performed *Trim26* knockdown in Raw264.7 cells and found a significant upregulation of key cell cycle genes, including *Cyclin D1*, *Cyclin A2*, *Cyclin E1*, and *Cyclin B1* (Fig. 4H), suggesting that *Trim26* knockdown promotes macrophage proliferation.

It has been reported that macrophage, including RAW264.7 cells, hepatic Kupffer cells and BMDM, have the ability to secrete TNF-α and IL-6 in the supernatants [29, 30]. These cytokines play significant roles in the initiation of the priming phase and increased G1/S transition in the hepatocyte cell cycle [31]. In our study, we also found that *Trim26* deficiency not only activated M1 polarization in BMDM, but also promoted the secretion of TNF-α and IL-6 (Fig. 4F). The levels of TNF-α and IL-6 were significantly increased in *Trim26*^−/−^ mice after PHx (Supplementary Fig. S4A). We also observed significantly increased mRNA levels of proliferative markers (*Cyclin D1, Cyclin E, Cyclin A2, Cyclin B1*) in IL-6-treated (10 ng/mL, 24 h) supernatants compared to non-stimulated cells (Supplementary Fig. S4B).

### M1 macrophage secrete Wnts to promote hepatic cells proliferation

**Fig. 5:**
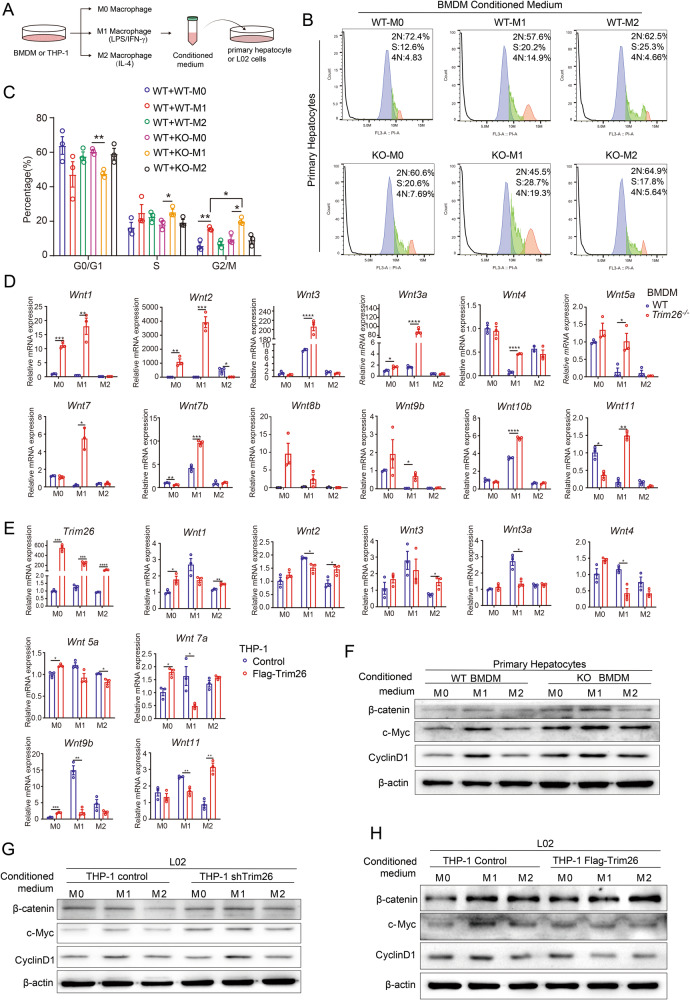
M1 macrophage of *Trim26*^−/−^ mice facilitates liver regeneration by secreting more Wnts. **A** Bone marrow-derived macrophages or THP-1 were induced M0, M1, and M2 polarization, respectively. Conditioned media were collected and applied to primary hepatocytes or L02 cells. **B**, **C** Primary hepatocytes were treated with different CMs, and cell cycle assay were measured by flow cryometry. **D** The relative expression of Wnts was detected in M0, M1, and M2 macrophages derived from the bone marrow of *Trim26*^*−/−*^ and WT mice. **E** Wnts mRNA expression was detected in M0, M1, and M2 macrophages from control and Flag-*Trim26* THP-1 cells. **F** Primary hepatocytes were treated with different CMs from BMDM, respectively. And the protein expression of β-catenin signaling proteins were measured. **G**, **H** L02 treated with CMs from THP-1, respectively, and the protein expression of β-catenin signaling proteins were measured. Representative data and average of triplicate repeats are shown. (*p < 0.05, **p < 0.01, ***p < 0.001, ****p < 0.0001).

Given that increased infiltration of M1 macrophages in *Trim26*^−/−^ mice, we then investigated the impact of these macrophages on liver cell proliferation. L02 cells or isolated mouse primary hepatocytes were stimulated with different types of conditional medium (CM) collected from cultured BMDMs or THP-1 cells (Fig. 5A). We observed that the conditioned medium from *Trim26*-deficient M1 macrophages enhanced the cell cycle progression of primary hepatocytes, as evidenced by flow cytometry analysis (Fig. 5B, C, Supplementary Fig. S5A).

We next wondered how exactly M1 macrophage regulates liver cell proliferation. The Wnts is mainly secreted by nonparenchymal cells, such as endothelial cells, macrophages [2], and the Wnt/β-catenin signaling pathway plays an important role in the process of liver regeneration [32]. Using BMDMs, we showed that *Trim26* knockout increased lipopolysaccharide (LPS)/IFN-γ-induced macrophage activation and the secretion of Wnts, such as *Wnt1, Wnt2, Wnt3, Wnt3a, Wnt4, Wnt5a, Wnt7b, Wnt8b, Wnt9b, Wnt10b, Wnt11*(Fig. 5D). The WB assays demonstrated that secretions from *Trim26*-deficient M1 macrophage potently activated the β-catenin signaling pathway (Fig. 5F). The THP-1 cells showed similar expression level of Wnts after treated with LPS/IFN-γ, and CMs from these cells also activated the Wnt signaling pathway on L02 cell (Fig. 5F, G). However, *Trim26* overexpression in THP-1 reversed M1 macrophages promoting liver proliferation (Fig. 5E, H). IWP-2 is an inhibitor that selectively blocks the secretion of Wnt proteins. Primary hepatocytes were stimulated with different types of CM collected from cultured Raw264.7 cells, with or without IWP-2 treatment. The treatment of Raw264.7 cells with IWP-2 reversed the function of M1 macrophages on the β-catenin signaling pathway in hepatocytes and inhibited liver cell proliferation (Supplementary Fig. S6A–C). These findings indicate that M1 macrophages with *Trim26* deficiency potently activated the β-catenin signaling to hepatocyte proliferation.

### *Trim26* deficiency in myeloid cells contribute to hepatocyte proliferation

**Fig. 6:**
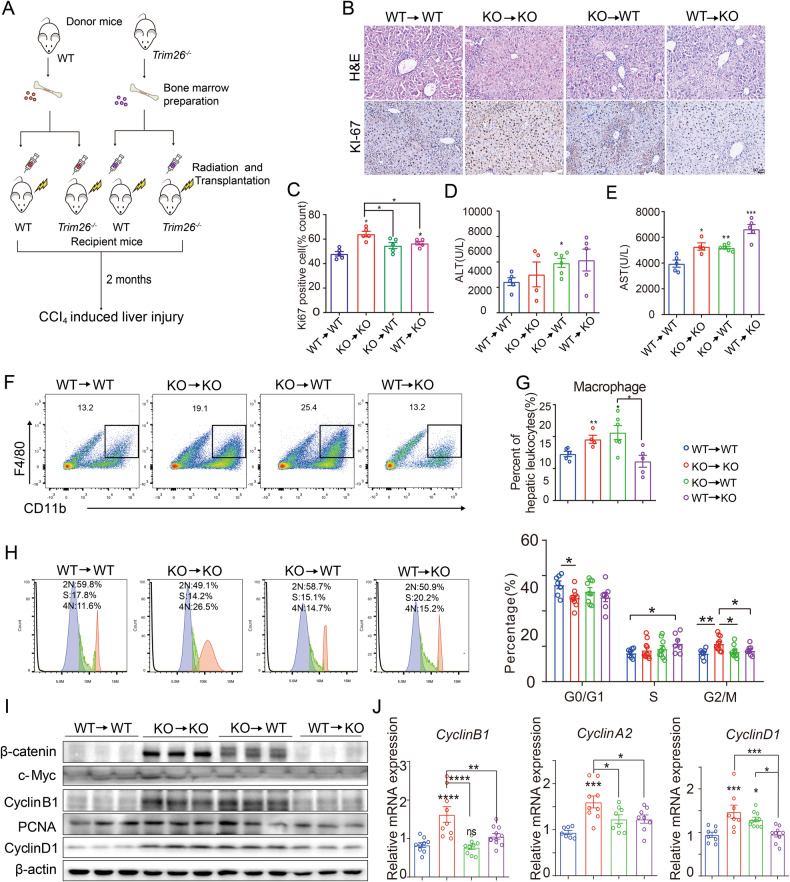
Bone marrow transplantation mitigated the promoting effect of *Trim26* deficiency on liver regeneration. **A** WT and *Trim26*^−/−^ mice were exposed to 8 Gy of γ radiation, and the bone marrow was injected into the mice through the tail vein. After 8 weeks, mice were treated with CCl_4_ for two days. **B**, **C** Representative HE staining and Ki67 staining and the statistical quantification. (*n* = 5, scale bar, 50 µm). **D**, **E** Serum ALT and AST levels. **F**, **G** Representative FACS plots and the statistical quantification of hepatic macrophage (F4/80^+^CD11b^+^) profiles of CD45^+^ liver monocytes from *Trim26*^−/−^ and WT mice on second day after CCl_4_ treatment. *n* = 4–6/group. **H** Representative graphs of flow cytometry analysis of primary hepatocytes cell cycle, and the statistical analysis of percentages of cells at different cell cycle stages (G0/G1, S and G2/M). *n* = 7–10/group. **I**, **J** mRNA levels and protein expression of cell cycle proteins and β-catenin signaling were examined after bone marrow transplantation. *n* = 7–10/group. (*p < 0.05, **p < 0.01, ***p < 0.001, ****p < 0.0001).

In order to investigate the potential role of *Trim26* deficiency in bone marrow-derived immune cells in the progression of liver regeneration, we isolated bone marrow from WT or *Trim26*^−/−^ mice and performed an in vivo experiment (Fig. 6A). Two months after the irradiation, flow cytometry confirmed the successful engraftment of macrophage bone marrow in the recipient mice. (Fig. 6F, G). Chimeric WT mice containing *Trim26*^−/−^ bone marrow (WT/*Trim26*^−/−^-BM) show a significant liver damage than chimeric WT mice containing WT bone marrow (WT/WT-BM), as evidenced by histological examination using H&E staining and measurement of serum levels of ALT and AST (Fig. 6B, D, E). Additionally, the promotion of CCl_4_-induced liver regeneration was significantly greater in WT/*Trim26*^−/−^-BM mice than in WT/WT-BM mice, as demonstrated by Ki67 staining, flow cytometry assays, WB, and qPCR assays (Fig. 6B, C, H–J). These findings suggest that TRIM26 deficient marrow-derived cells enhance liver cell proliferation compared to WT marrow-derived cells. More importantly, *Trim26*^−/−^/WT-BM mice also potently promoted CCl_4_-induced liver regeneration compared with WT/WT-BM mice (Fig. 6B, C, H–J). Therefore, these results indicate that *Trim26* deficiency both in bone marrow-derived cells and hepatocytes prompt liver cell regeneration through different mechanisms.

### The deletion of *Trim26* promotes hepatocyte proliferation through the Wnt/β-catenin signaling pathway

**Fig. 7:**
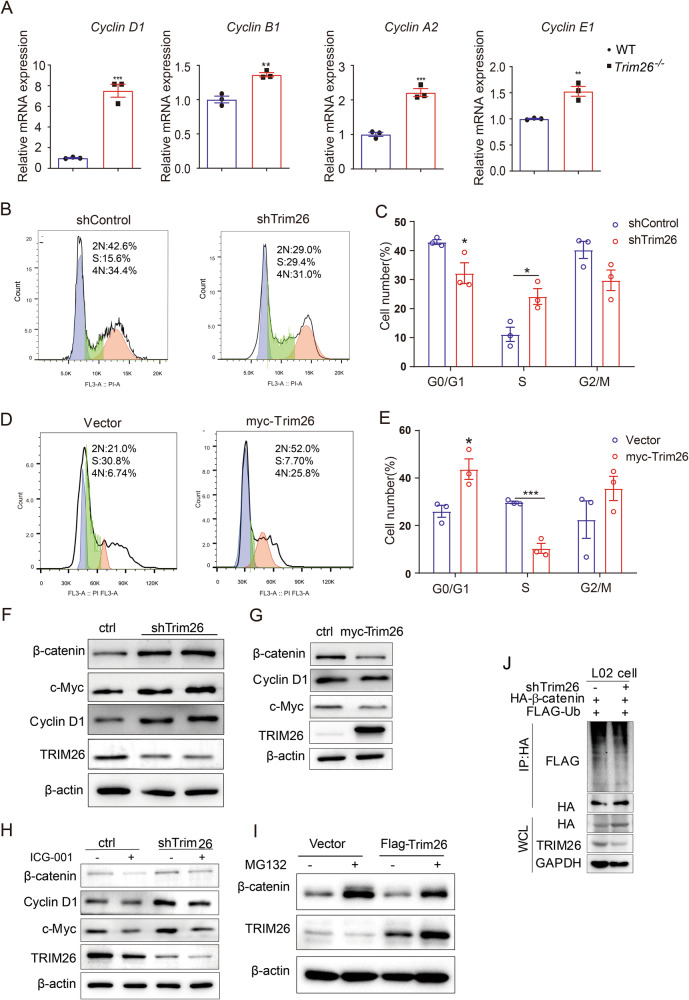
*Trim26* deficiency in hepatocyte promotes hepatocyte proliferation through Wnt/β-catenin signaling pathway. **A** Primary hepatocytes were cultured in complete medium for 24 h, then the relative expression of cell cycle proteins was detected. **B**–**E** Representative graphs of flow cytometry analysis of L02 cell cycle, and the statistical analysis of percentages of cells at different cell cycle stages (G0/G1, S and G2/M). **F** L02 cells were transfected with *Trim26* shRNA, and cell lysates were subjected to WB. **G** L02 cells were transfected with *Trim26*, and cell lysates were subjected to WB. **H** L02 cells were transfected with *Trim26* shRNA. After treatment with or without ICG-001 (10 µM/mL) for 24 h, cell lysates were subjected to WB. **I** L02 cells were transfected with FLAG- *Trim26*. After treatment with or without MG132 (10 µM/mL) for 6 h, cell lysates were subjected to WB. **J** Representative immunoblotting bands showing the indicated proteins in the sh*Trim26* and shControl L02 cells transfected with HA-tagged ubiquitin, Flag-β-catenin, and MG132 treatment. (*p < 0.05, **p < 0.01, ***p < 0.001).

It is noteworthy to mention that primary isolated hepatocytes from *Trim26*^*−/−*^ mice exhibited an increase in cyclin protein expression compared to WT mice, consistent with the results of adding HGF (Fig. 7A, Supplementary Fig. S7A), indicating that the lack of TRIM26 in hepatocytes promotes cell cycle progression. To validate this hypothesis, we performed a cell cycle assay. As depicted in Fig. 7B–E, *Trim26* knockdown resulted in an increased transition from G1 to S phase, while the overexpression of *Trim26* led to a decreased G1 to S phase transition. These findings suggest that *Trim26* acts as a suppressor of DNA replication during the S phase. Furthermore, *Trim26* knockdown increased the expression of β-catenin, c-Myc and CyclinD1. In contrast, overexpression of *Trim26* led to a reduction in the β-catenin signaling pathway (Fig. 7F, G). Meanwhile, treatment of with the Wnt/β-catenin inhibitor ICG-001 effectively counteracted the promotional effects observed in the *Trim26* knockdown condition (Fig. 7H). In liver cancer, the degradation of β-catenin occurs through ubiquitination, specifically mediated by TRIM26 [17]. As anticipated, the upregulation of β-catenin was dramatically reversed in TRIM26 knockdown L02 cells treated with MG132 (Fig. 7I). Additionally, the immunoprecipitation assay revealed that TRIM26 exerted a positive effect on the ubiquitination process of β-catenin in L02 cells (Fig. 7J). Altogether, these results indicate that the deletion of *Trim26* promotes hepatocyte proliferation by facilitating the β-catenin signaling pathway.

**Fig. 8:**
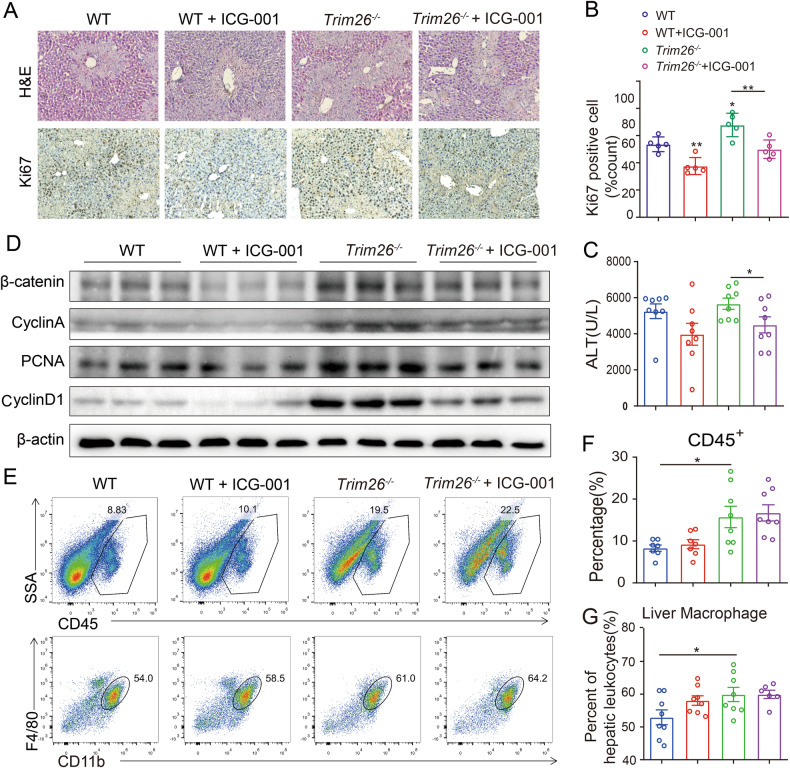
ICG-001 represses liver regeneration through the β-catenin signaling pathway in CCl_4_ induced liver injury. **A**, **B** Representative HE staining and Ki67 staining and the statistical quantification. (*n* = 5, scale bar, 50 µm). **C** Serum ALT levels. **D** Protein expression of cell cycle proteins and β-catenin signaling with ICG-001 treatment. **E**–**G** Representative FACS plots and statistical quantification of hepatic macrophages (F4/80^+^CD11b^+^) and CD45^+^ liver monocytes. *n* = 6–8/group. (*p < 0.05, **p < 0.01).

Given that *Trim26* deficiency promotes liver regeneration through Wnt/β-catenin pathway, we next determined whether targeting β-catenin could block the hepatocyte proliferation caused by *Trim26* depletion. As shown in Fig. 8A, C mice showed reduction of liver injury after ICG-001 treatment, as evidenced by a reduction of lobular inflammation and necrosis, and a potent reduction of plasma levels of ALT. As shown in Fig. 8A, B, D, the inhibition of β-catenin demonstrated a notable decrease in hepatocyte proliferation, as well as a down-regulated β-catenin signaling, as confirmed by decreased Ki67 staining and Western blot assay. However, β-catenin inhibition had no discernible impact on macrophage infiltration (Fig. 8C–E). These findings suggest the potential of targeting TRIM26 to promote hepatocytes proliferation after hepatectomy.

## Discussion

The capacity for liver regeneration holds significant importance in individuals who have undergone resection for hepatocellular carcinoma or served as liver transplant donors. Therefore, it is imperative to understand the process and molecular mechanisms of liver regeneration to minimize the risk of postoperative liver failure. TRIM26, an E3 ubiquitin ligase, is considered as a key regulator of protein degradation by ubiquitination. TRIM26 has been found to be downregulated in various types of cancers [19, 33] and has been shown to impede the progression of hepatocellular carcinoma [21]. In this study, we identified the E3 ubiquitin ligase TRIM26 as a novel negative regulator of liver regeneration in mice following partial hepatectomy or acute liver injury induced by CCl_4_.

Our results demonstrate that *Trim26* deficiency enhances hepatocyte proliferation and liver regeneration through two distinct but interconnected mechanisms. First, *Trim26* knockout promotes the recruitment of macrophages to the liver and their polarization towards a pro-inflammatory M1 phenotype while hindering M2 polarization. The M1 macrophages secrete pro-inflammatory factors and Wnts, which stimulate hepatocyte proliferation by activation the Wnt/β-catenin signaling pathway. Second, in hepatocytes, the deletion of *Trim26* reduces the ubiquitination and degradation of β-catenin, further enhancing Wnt/β-catenin signaling and driving cell cycle progression. The pro-regenerative effects of *Trim26* deficiency are attenuated by pharmacological inhibition of the Wnt/β-catenin pathway or macrophage depletion, underscoring the functional significance of these mechanisms.

Our findings are consistent with previous studies highlighting the critical role of macrophages in liver regeneration. For instance, macrophage-derived Wnt3a has been shown to promote hepatocyte proliferation during liver regeneration in mice [34]. Moreover, macrophage depletion impairs liver regeneration after partial hepatectomy [35]. Inflammation plays a crucial role in the regeneration and remodeling of damaged areas, serving a protective function by eliminating cellular debris and promoting regeneration [3]. In the current study, *Trim26*-deficient macrophages exhibit an increased secretion of IL-6 and TNF-α. TNF-α has been shown to facilitate the process of regeneration while also inhibiting apoptosis. Inflammatory molecules, such as IL-6, are produced within 6 h after hepatectomy to initiate liver regeneration. IL-6 increases inflammation, exacerbating injury on the one hand, and promotes proliferation on the other. IL-6 activates classical signaling through the formation of membrane-bound IL-6R and gp130 complexes, thereby facilitating liver regeneration [36]. In the present study, we also demonstrated that *Trim26* deficiency resulted in enhanced secretion of IL-6 by macrophages, which in turn promoted liver regeneration. Macrophage recruited from the bone marrow have been shown to accelerate liver regeneration after partial hepatectomy [35]. Vagal signaling-mediated production of IL-6 in hepatic macrophages plays a crucial role in liver regeneration and ensures survival [37]. Our study extends these findings by identifying TRIM26 as a key regulator of macrophage polarization and function in the context of liver regeneration.

The Wnt/β-catenin signaling pathway is a well-established driver of liver regeneration. Wnt2 has been shown to promote lung adenocarcinoma and fibroblast proliferation [38, 39]. In the present study, we found that deletion of *Trim26* led to a significant increase in the secretion of Wnt2, reaching levels several orders of magnitude higher than the control. Wnt2 was associated with metabolic zonation and liver regeneration [14]. Delayed secretion of Wnts has been found to result in delayed liver regeneration [2]. In the classical Wnt/β-catenin signaling pathway, the removal of Wnts triggers the degradation of β-catenin through the formation of a degradation complex involving Axin, APC, GSK3, and CK1. Conversely, Wnts ligands are able to inhibit β-catenin degradation, resulting in the accumulation of β-catenin in the nucleus. This accumulation facilitates the formation of a complex between β-catenin and the TCF/LEF family of transcription factors, subsequently activating the transcription of downstream target genes. Secondly, the regulation of intracellular β-catenin is also governed by the ubiquitin-proteasome pathway-regulated β-catenin destruction complex and β-catenin degradation [40]. Our data indicate that TRIM26 negatively regulates this pathway through two complementary mechanisms: by modulating the polarization of macrophages, which are a major source of Wnts in the regenerating liver, and by directly promoting the ubiquitination and degradation of β-catenin in hepatocytes. These findings are in line with our previous work demonstrating that TRIM26 inhibits hepatocellular carcinoma progression by targeting β-catenin for degradation [17].

While our study provides novel insights into the role of TRIM26 in liver regeneration, it also has several limitations. First, we primarily relied on global *Trim26* knockout mice and did not fully elucidate the cell-type-specific functions of TRIM26 in the liver. Although our bone marrow transplantation experiments suggest that myeloid cell *Trim26* is important for liver regeneration, the contribution of hepatocyte TRIM26 remains to be fully explored. Second, while we focused on the Wnt/β-catenin pathway, other signaling cascades may also be involved in mediating the effects of TRIM26 on liver regeneration. Future studies using cell-type-specific knockout models and unbiased approaches such as RNA sequencing will be necessary to address these questions.

## Conclusion

In summary, our study identifies TRIM26 as a negative regulator of liver regeneration that acts by modulating macrophage polarization and Wnt/β-catenin signaling in hepatocytes. *Trim26* deficiency increases the number of M1 macrophage and promotes M1 macrophage polarization to secrete more Wnts, thereby activating the Wnt/β-catenin pathway. In hepatocytes, loss of TRIM26 reduces the ubiquitination and degradation of β-catenin, further enhancing Wnt/β-catenin signaling and driving liver cell proliferation. Pharmacological and genetic strategies aimed at modulating the activity of TRIM26 have the potential to enhance liver regeneration in cases of severe liver damage and improve the overall clinical outcome of liver transplantation.

### Supplementary information

Supplementary figures

WB

## Data Availability

All data and materials have been made available.
