# Supplementary material for: TRIM26 deficiency enhancing liver regeneration through macrophage polarization and β-catenin pathway activation
Source: Cell Death Dis. 2024 Jun 26;15(6):453. doi: 10.1038/s41419-024-06798-0 (PMC11208526; doi:10.1038/s41419-024-06798-0)
Supplement: Supplementary file 1 — Supplementary figures [file 41419_2024_6798_MOESM1_ESM.docx]

**Table S1**: Primer sequences for the amplification

| Gene Name | Primer Sequence（5′→3′） |
| --- | --- |
| ms-*TGM2*-F | GCTGGACCAACAGGACAATGT |
| ms-*TGM2*-R | CTCTAGGCTGAGACGGTACAG |
| ms-*Wnt10b*-F | GCGGGTCTCCTGTTCTTGG |
| ms-*Wnt10b*-R | CCGGGAAGTTTAAGGCCCAG |
| ms-*Wnt7b*-F | CTTCACCTATGCCATCACGG |
| ms-*Wnt7b*-R | TGGTTGTAGTAGCCTTGCTTCT |
| ms-*Wnt9b*-F | ACCTGAAGCAGTGTGACCTAC |
| ms-*Wnt9b*-R | GCTCCTGCCTGAACTGGAA |
| ms-*GAPDH*-F | TGACCTCAACTACATGGTCTACA |
| ms-*GAPDH*-R | CTTCCCATTCTCGGCCTTG |
| ms-*Wnt5a*-F | CAACTGGCAGGACTTTCTCAA |
| ms-*Wnt5a*-R | CCTTCTCCAATGTACTGCATGTG |
| ms-*Wnt8b*-F | CCCGTGTGCGTTCTTCTAGTC |
| ms-*Wnt8b*-R | GTAGACCAGGTAAGCCTTTGGA |
| ms-*Wnt1*-F | GGTTTCTACTACGTTGCTACTGG |
| ms-*Wnt1*-R | GGAATCCGTCAACAGGTTCGT |
| ms-*Wnt3a*-F | CTCCTCTCGGATACCTCTTAGTG |
| ms-*Wnt3a*-R | CCAAGGACCACCAGATCGG |
| ms-*Wnt3*-F | AGCGTAGCAGAAGGTGTGAAG |
| ms-*Wnt3*-R | CCAGGTGGCCCCTTATGATG |
| ms-*Wnt11*-F | GCACTGAATCAGACGCAACAC |
| ms-*Wnt11*-R | CGACAGGGCATACACGAAGG |
| ms-*Wnt9b*-F | ACCTGAAGCAGTGTGACCTAC |
| ms-*Wnt9b*-R | GCTCCTGCCTGAACTGGAA |
| ms-*Cyclin A2*-F | GCCTTCACCATTCATGTGGAT |
| ms-*Cyclin A2*-R | TTGCTCCGGGTAAAGAGACAG |
| ms-*Cyclin E1*-F | CCGTCTTGAATTGGGGCAATA |
| ms-*Cyclin E1*-R | GAGCTTATAGACTTCGCACACC |
| ms-*Wnt2*-F | CCTGATGAACCTTCACAACAAC |
| ms-*Wnt2*-R | TCTTGTTTCAAGAAGCGCTTTAC |
| ms-*Wnt4*-F | ACTGGACTCCCTCCCTGTCT |
| ms-*Wnt4*-R | TGCCCTTGTCACTGCAAA |
| ms-*TGF-β*-F | TGGATGGGTTACAGGTTACACA |
| ms-*TGF-β*-R | CCAGTTGCGAAAGCCGAAA |
| ms-*IL-6*-F | GGCGGATCGGATGTTGTGAT |
| ms*-IL-6*-R | GGACCCCAGACAATCGGTTG |
| ms-*CyclinB1*-F | CTTACTCTGCGCTCCGAAGG |
| ms-*CyclinB1*-R | TTGGACATGCTGGTGAGGTT |
| ms-*Cyclin D1*-F | GCGTACCCTGACACCAATCTC |
| ms-*Cyclin D1*-R | ACTTGAAGTAAGATACGGAGGGC |
| ms-*TNF-α*-F | GGAACACGTCGTGGGATAATG |
| ms-*TNF-α*-R | GGCAGACTTTGGATGCTTCTT |
| ms-*IL-1α*-F | TGAAAACACAGAAGTAACGTCCG |
| ms-*IL-1α*-R | CCCAGGAGGAAATTGTAATGGGA |
| ms-*iNOS*-F | CTCTTCGACGACCCAGAAAAC |
| ms-*iNOS*-R | CAAGGCCATGAAGTGAGGCTT |
| ms-*CD86*-F | TCAATGGGACTGCATATCTGCC |
| ms-*CD86*-R | GCCAAAATACTACCAGCTCACT |
| ms-*Fizzl*-F | GAGATGCGGAGAACCCTGAC |
| ms-*Fizzl*-R | TGGAAGTTCACGCTCCAGTTG |
| ms-*Wnt7*-F | CTTCACCTATGCCATCACGG |
| ms-*Wnt7*-R | TGGTTGTAGTAGCCTTGCTTCT |
| ms-*Wnt7a*-F | GGCTTCTCTTCGGTGGTAGC |
| ms-*Wnt7a*-R | TGAAACTGACACTCGTCCAGG |


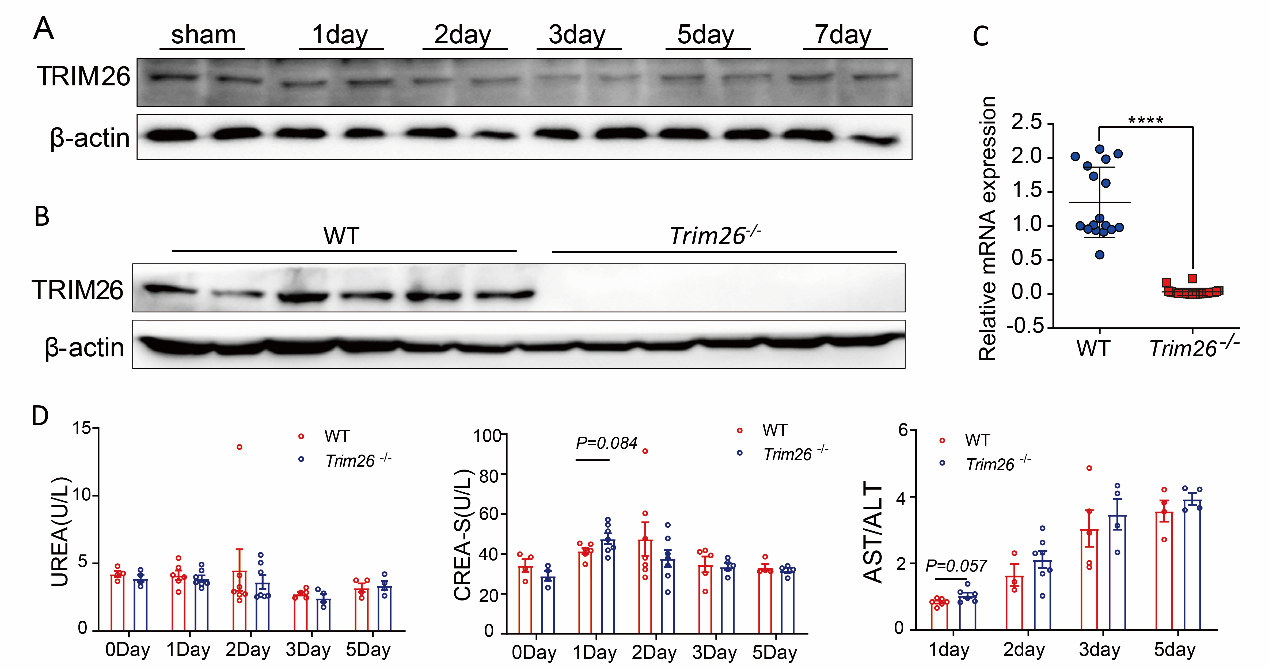


**Fig. S1** TRIM26 expression negatively correlates with liver proliferation after PHx. (A)The protein levels of TRIM26 in the livers of sham-operated mice and hepatectomized mice after PHx at different time points (1D–7D) were determined by western blotting. (B and C) The mRNA and protein levels of TRIM26 in *Trim26*^-/-^ and WT mice were determined by western blotting and qRT-PCR. n=17-20/group. (D) The UREA and CREA-S in the livers of sham-operated mice and hepatectomized mice after PHx at different time points (1day–7day) were determined. n=4-7/group. （*****p*<0.0001）


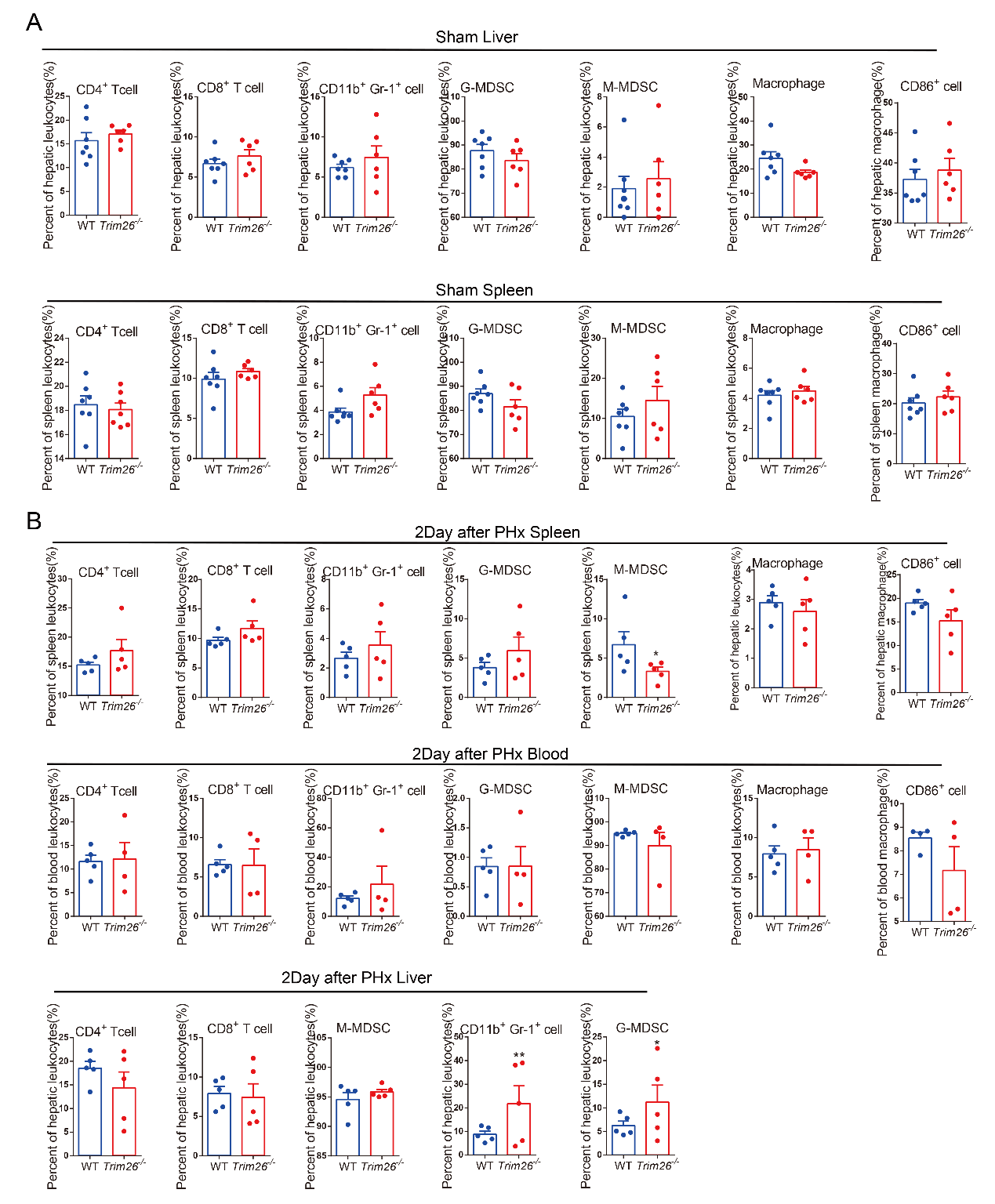


**Fig. S2** *Trim26* knockout promotes macrophages recruitment in liver

(A, B) Hepatic leukocytes populations were assessed by FACS analysis. The gating strategy to identify, CD8^+^ T cells (CD45^+^ CD8^+^), CD4^+^ T cells (CD45^+^ CD4^+^), hepatic macrophages (F4/80^+^CD11b^+^) G-MDSCs（CD11b^+^Ly6G^+^）, and M-MDSCs (CD11b^+^Ly6C^+^) gating from CD45^+^ cells are shown in *Trim26*^-/-^ and WT mice at sham or 2days after PHx are quantified as a proportion of total liver leukocytes. n=4-7/group. (**p*<0.05, ***p*<0.01).


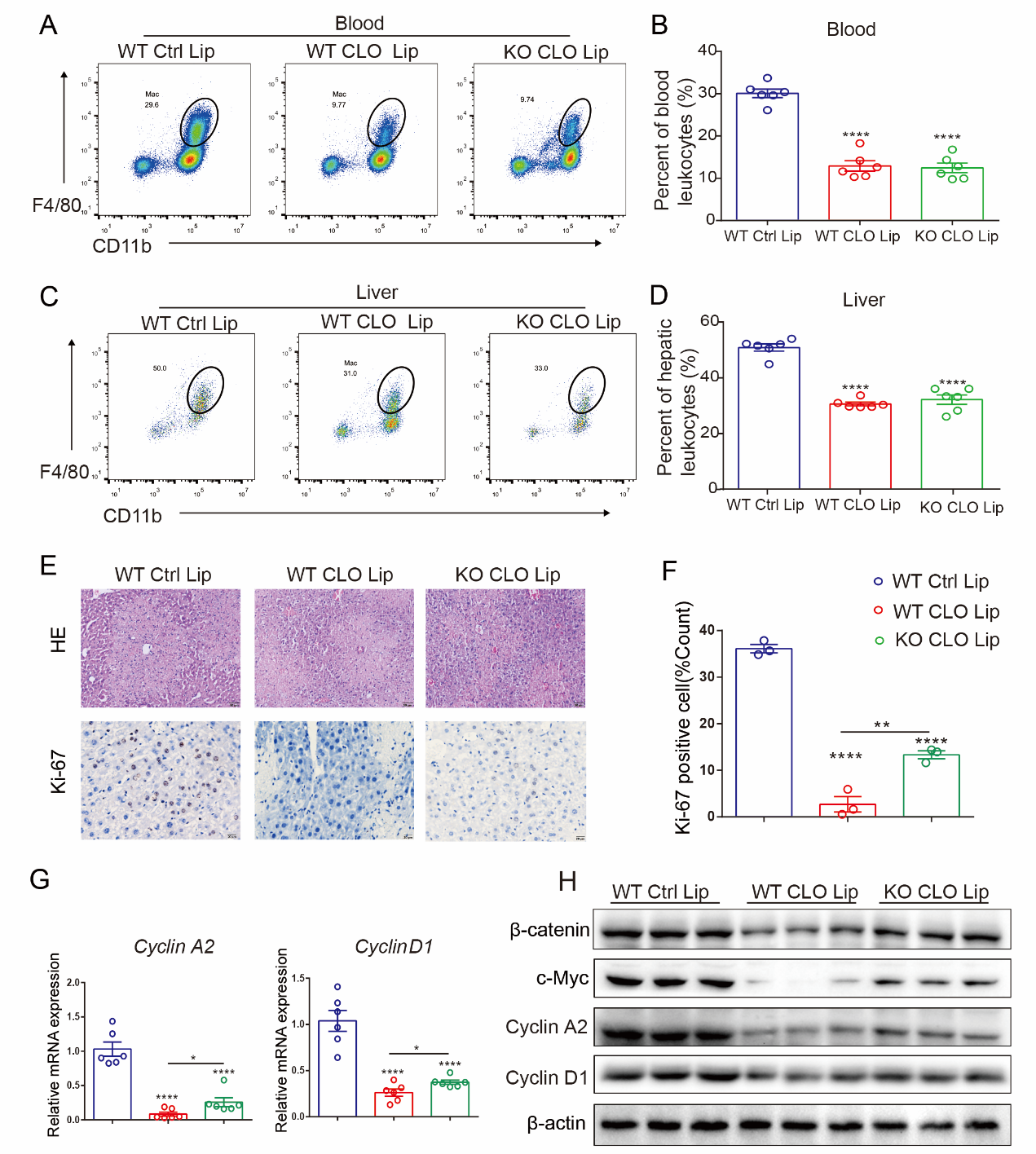


**Fig. S3** Macrophage depletion represses liver regeneration in CCl_4_ induced liver injury

(A-D) In vivo administration of PBS or clodronate liposome established macrophage depletion model. Liver and blood macrophages populations were assessed by FACS analysis and the statistical quantification. (E-F) Representative HE staining and Ki67 staining and the statistical quantification. (n=3, scale bar, 50 µm or 20 µm). (G) The relative mRNA expression of cell cycle proteins was detected by qPCR after clodronate liposome treatment. (H) Protein expression of cell cycle protein and β-catenin signaling with clodronate liposome treatment. (**p*<0.05, ***p*<0.01, *****p*<0.0001)


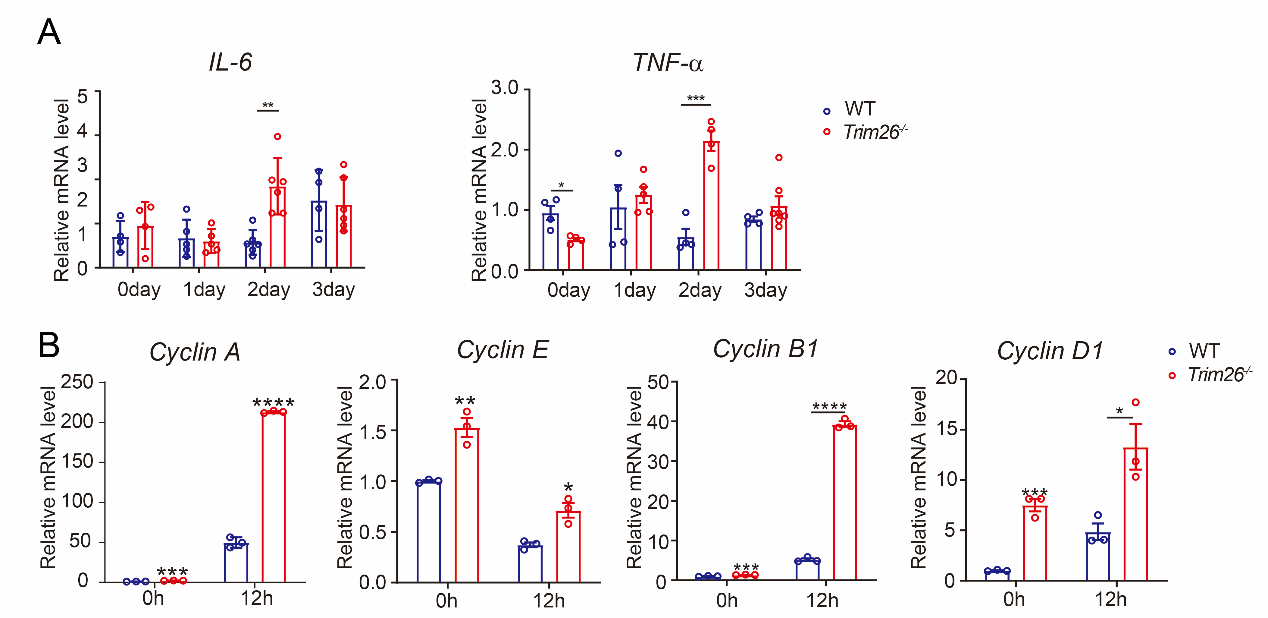


**Fig. S4** *Trim26*^-/-^ Increased the level of IL-6 and it promotes hepatocyte proliferation.

(A) the levels of *IL-6* and *TNF-α* in liver were detected by qPCR after PHx. n=4-7/group. (B) Primary hepatocytes treated with IL-6 (10ng/mL) for 12h, the relative expression of cell cycle protein was detected. (**p*<0.05, ***p*<0.01, ****p*<0.001, *****p*<0.0001).


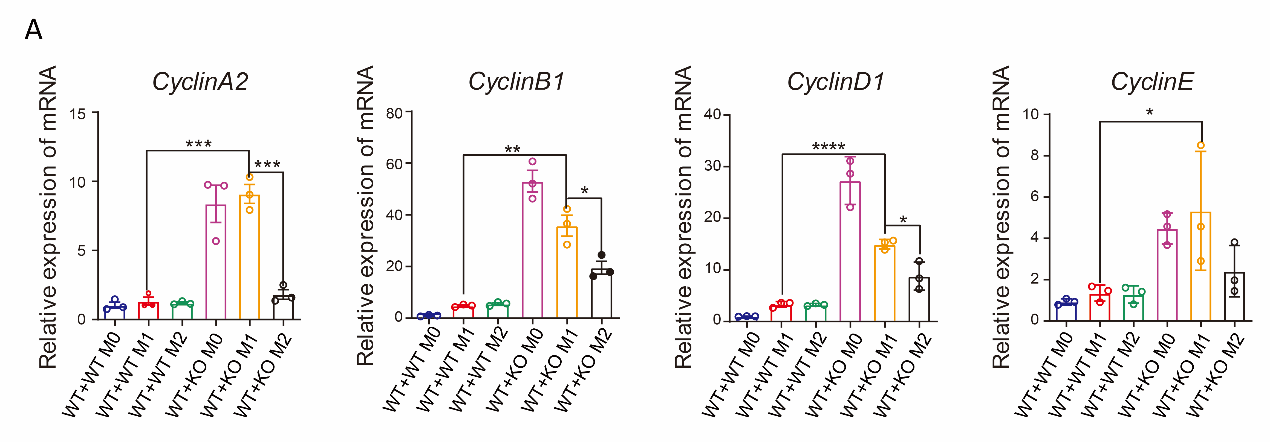


**Fig. S5** (A)BMDM were induced M0, M1, and M2 polarization, respectively. Conditioned media were collected and applied to primary hepatocytes. The relative mRNA expression of cell cycle proteins was detected. (**p*<0.05, ***p*<0.01, ****p*<0.001, *****p*<0.0001).


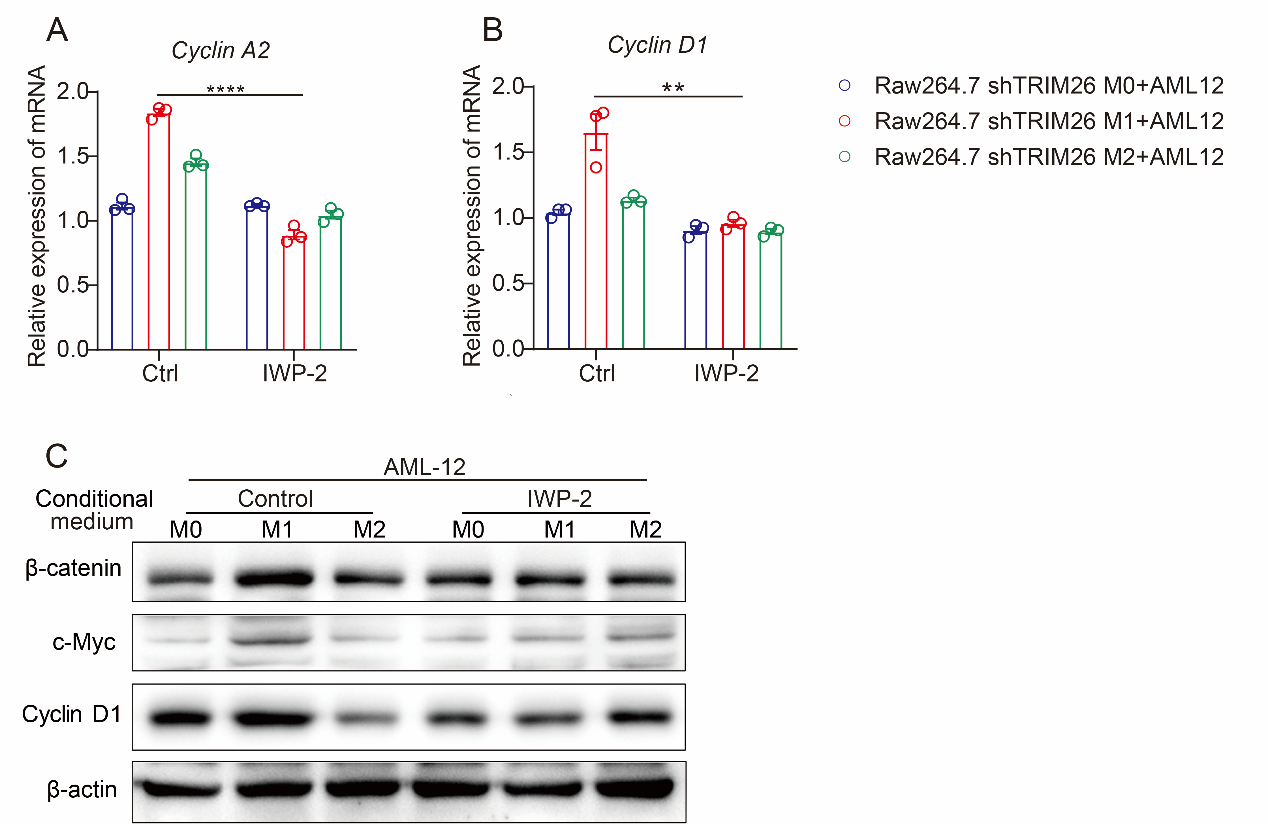


**Fig. S6** Raw264.7 cells with *Trim26* knockdown were induced M0, M1, and M2 polarization. Afterward, the Raw264.7 cells were treated with IWP-2 for 24 hours. Conditioned media were collected and applied to primary hepatocytes. (A-B) The relative mRNA expression of cell cycle proteins was detected. (C) The protein expression of β-catenin signaling. (***p*<0.01, *****p*<0.0001).


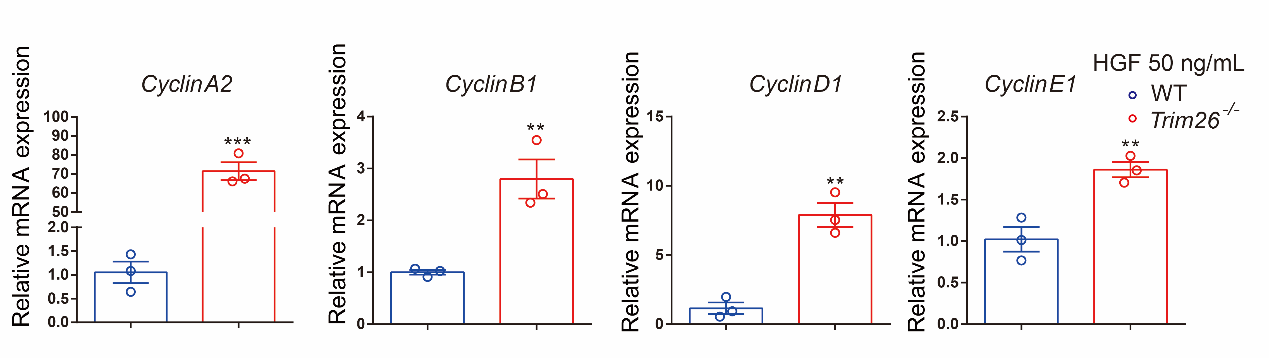


**Fig. S7** (A) Primary hepatocytes were cultured in a medium containing 2% FBS and 50 ng/mL HGF for 24 hours. Subsequently, the relative mRNA expression of cell cycle proteins was detected. (***p*<0.01, ****p*<0.001).
