# Supplementary material for: TRIM26 deficiency enhancing liver regeneration through macrophage polarization and β-catenin pathway activation
Source: Cell Death Dis. 2024 Jun 26;15(6):453. doi: 10.1038/s41419-024-06798-0 (PMC11208526; doi:10.1038/s41419-024-06798-0)
Supplement: Supplementary file 2 — WB [file 41419_2024_6798_MOESM2_ESM.pdf]

Fig 1G

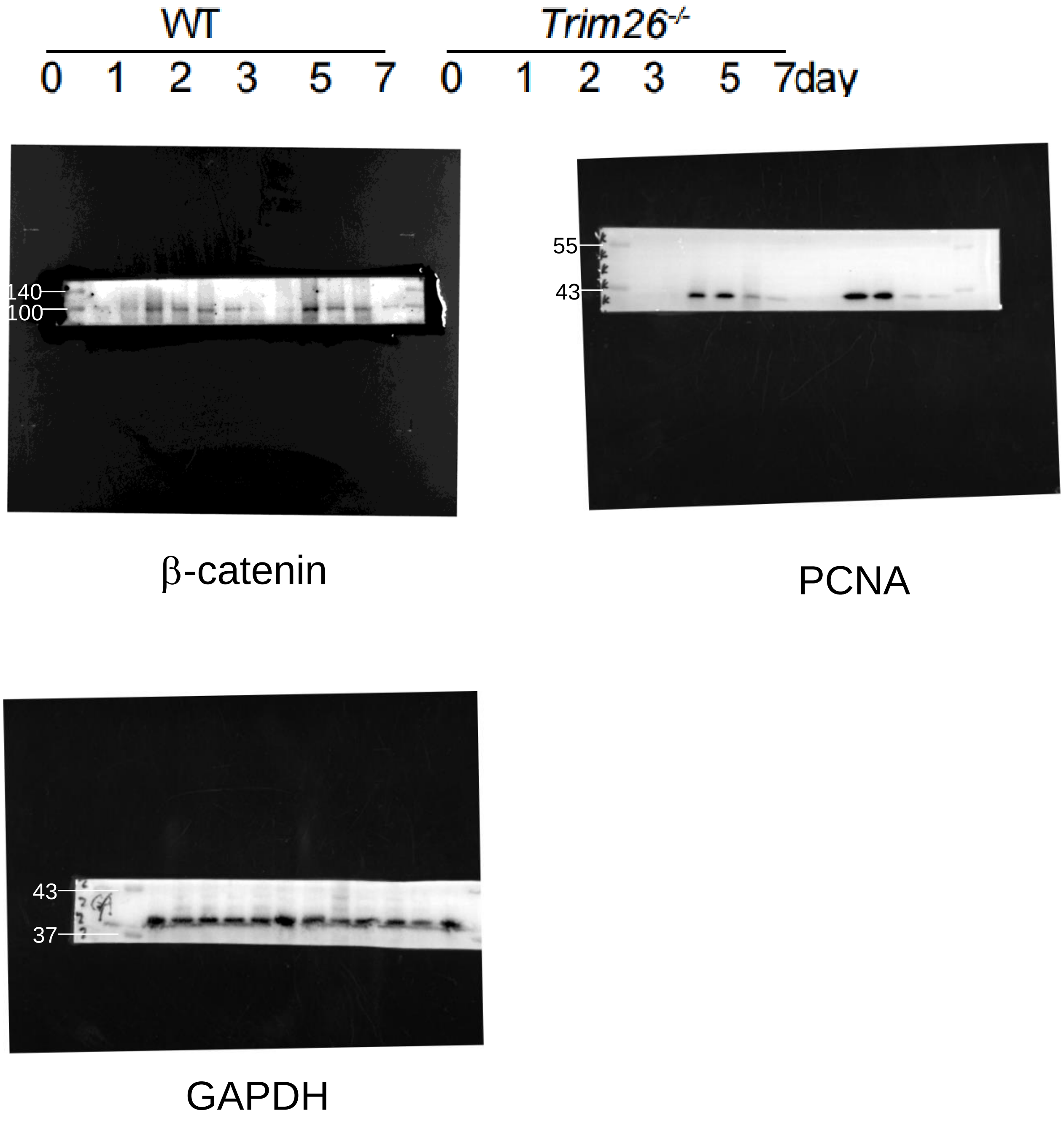
WT
Trim26$^{-/-}$
0 1 2 3 5 7 0 1 2 3 5 7day
140
100
55
43
43
37
β-catenin
PCNA
GAPDH

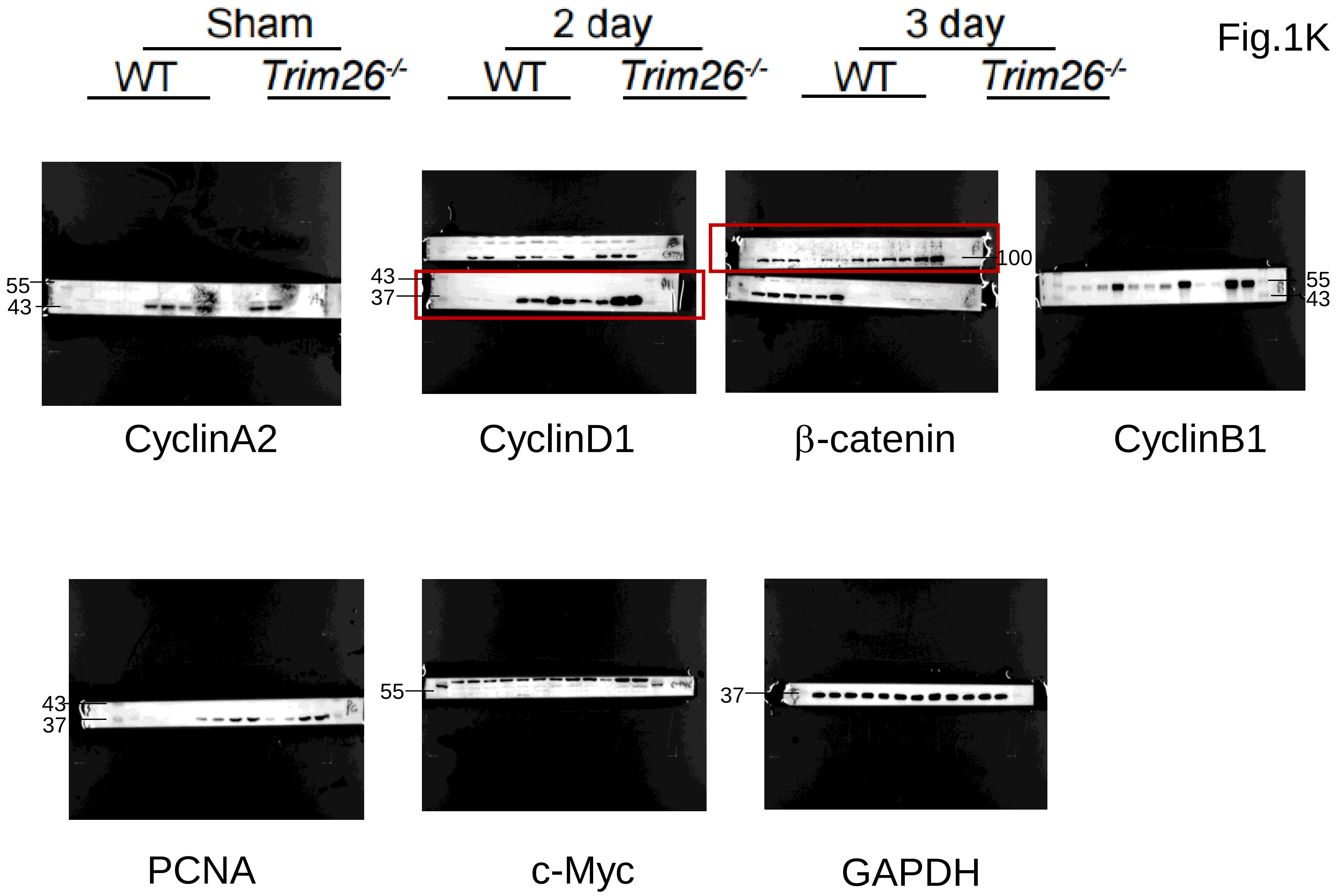
Sham
2 day
3 day
WT
Trim26-/-
WT
Trim26-/-
WT
Trim26-/-
Fig.1K
55
43
43
37
100
55
43
CyclinA2
CyclinD1
β-catenin
CyclinB1
43
37
55
37
PCNA
c-Myc
GAPDH

Fig.2D

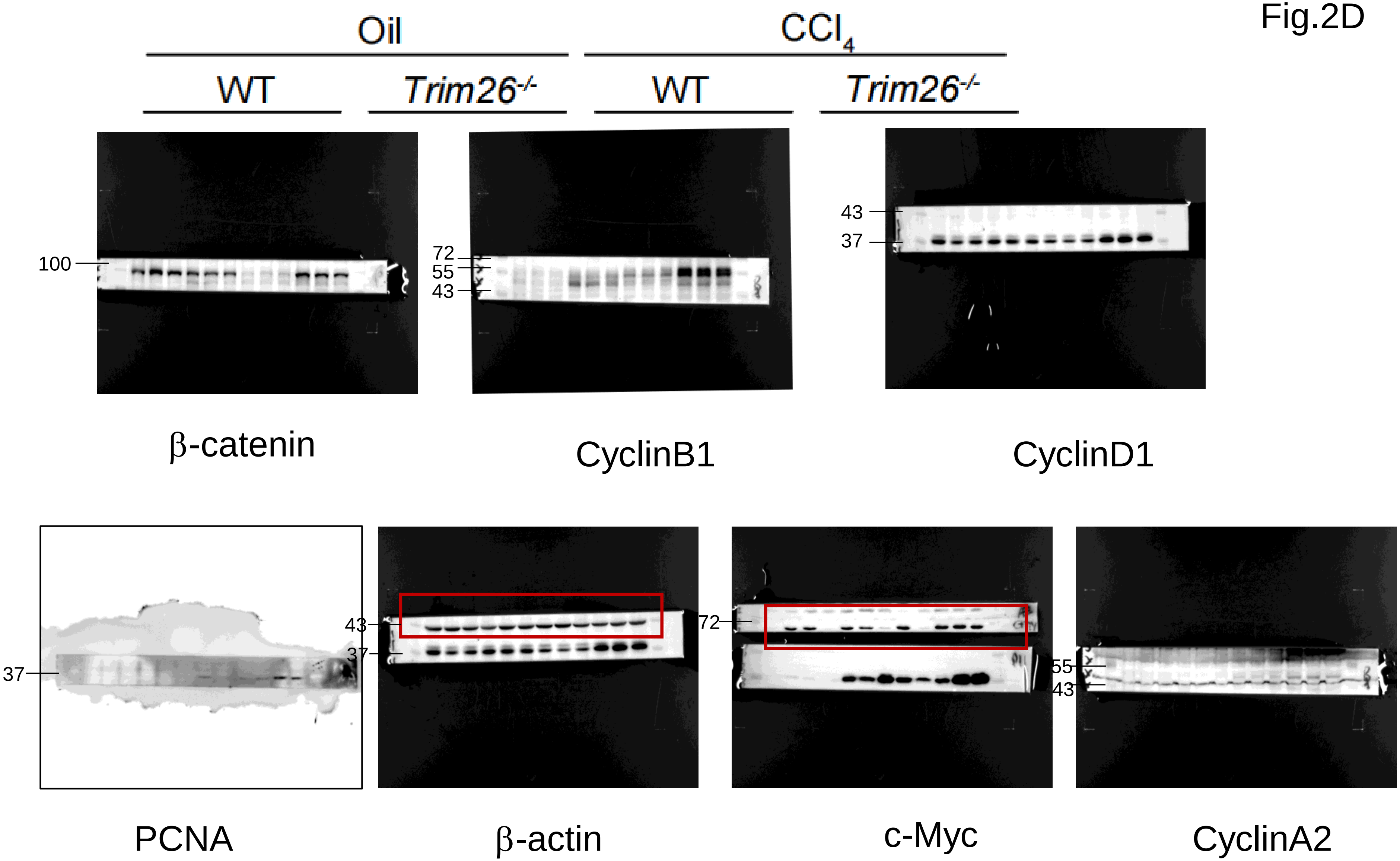

Oil
CCl4
WT
Trim26-/-
WT
Trim26-/-
100
72
55
43
43
37
β-catenin
CyclinB1
CyclinD1
37
43
37
72
55
43
PCNA
β-actin
c-Myc
CyclinA2

Fig.3I AAV

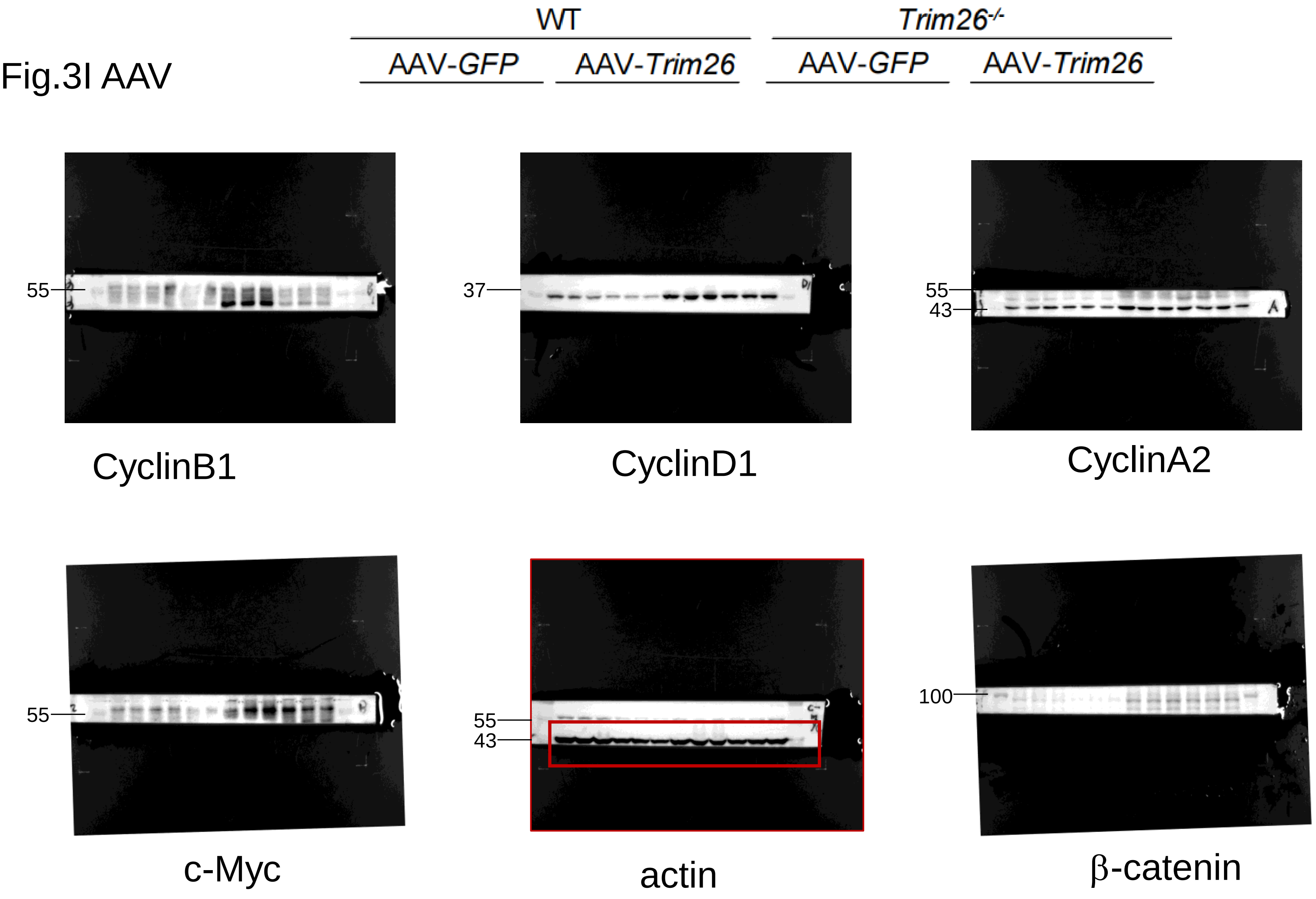
WT
Trim26-/-
AAV-GFP
AAV-Trim26
AAV-GFP
AAV-Trim26
55
CyclinB1
37
CyclinD1
55
43
CyclinA2
55
c-Myc
55
43
actin
100
β-catenin

FIG 5F

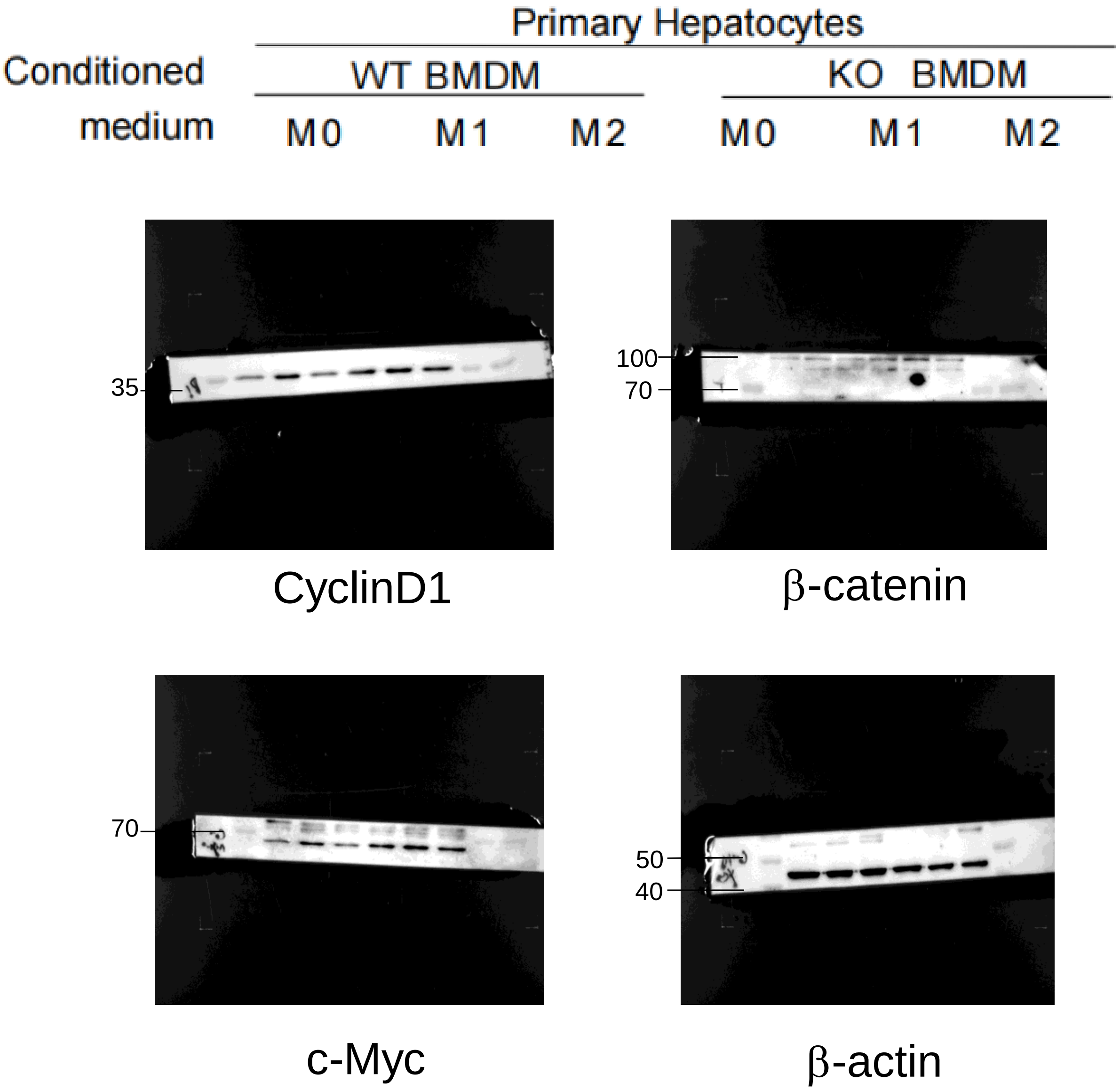
Primary Hepatocytes
Conditioned medium
WT BMDM
KO BMDM
M0
M1
M2
M0
M1
M2
35
100
70
CyclinD1
β-catenin
70
50
40
c-Myc
β-actin

Fig 5G

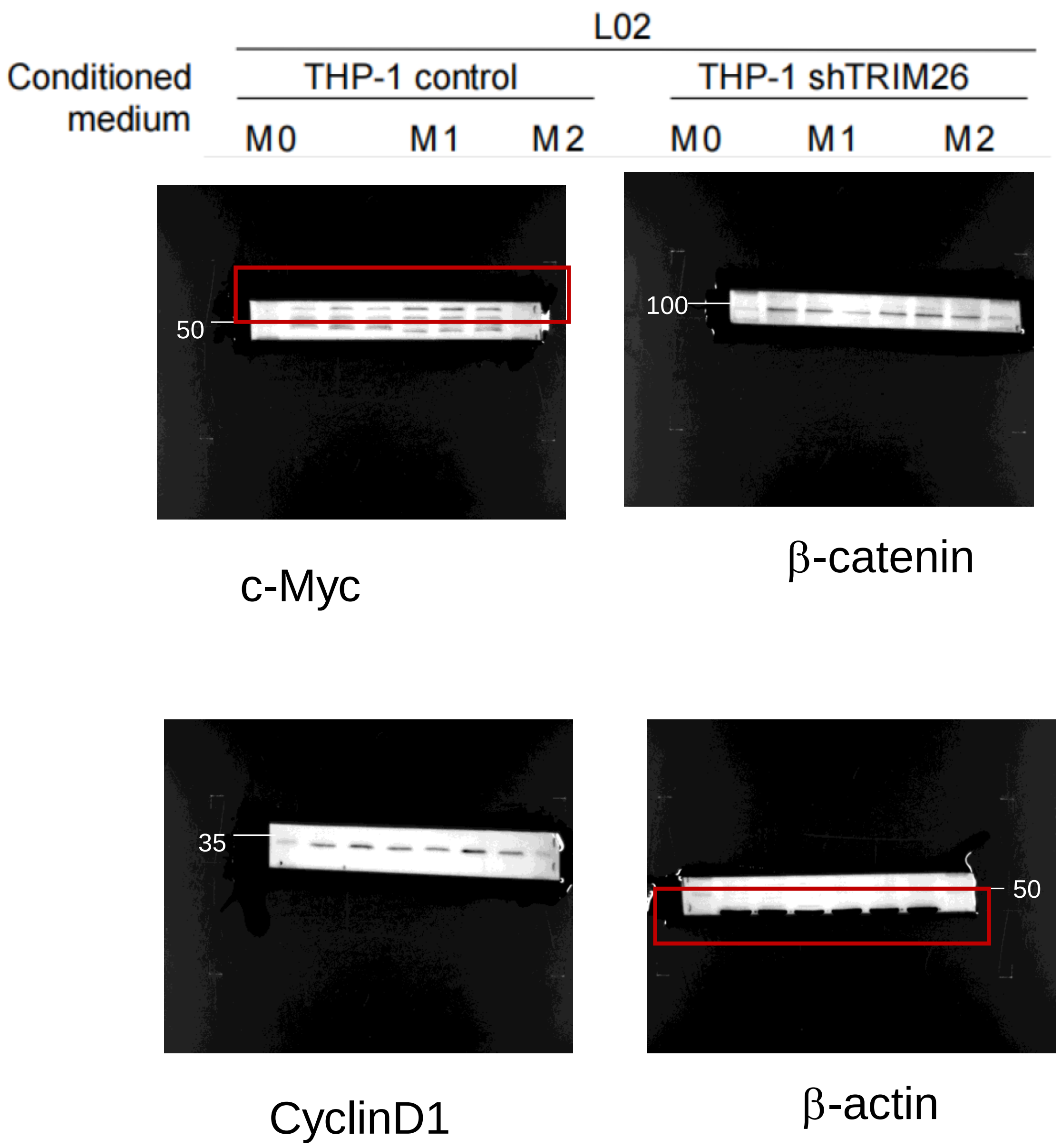
L02
Conditioned medium
THP-1 control
THP-1 shTRIM26
M0
M1
M2
M0
M1
M2
50
100
c-Myc
β-catenin
35
50
CyclinD1
β-actin

Fig 5H

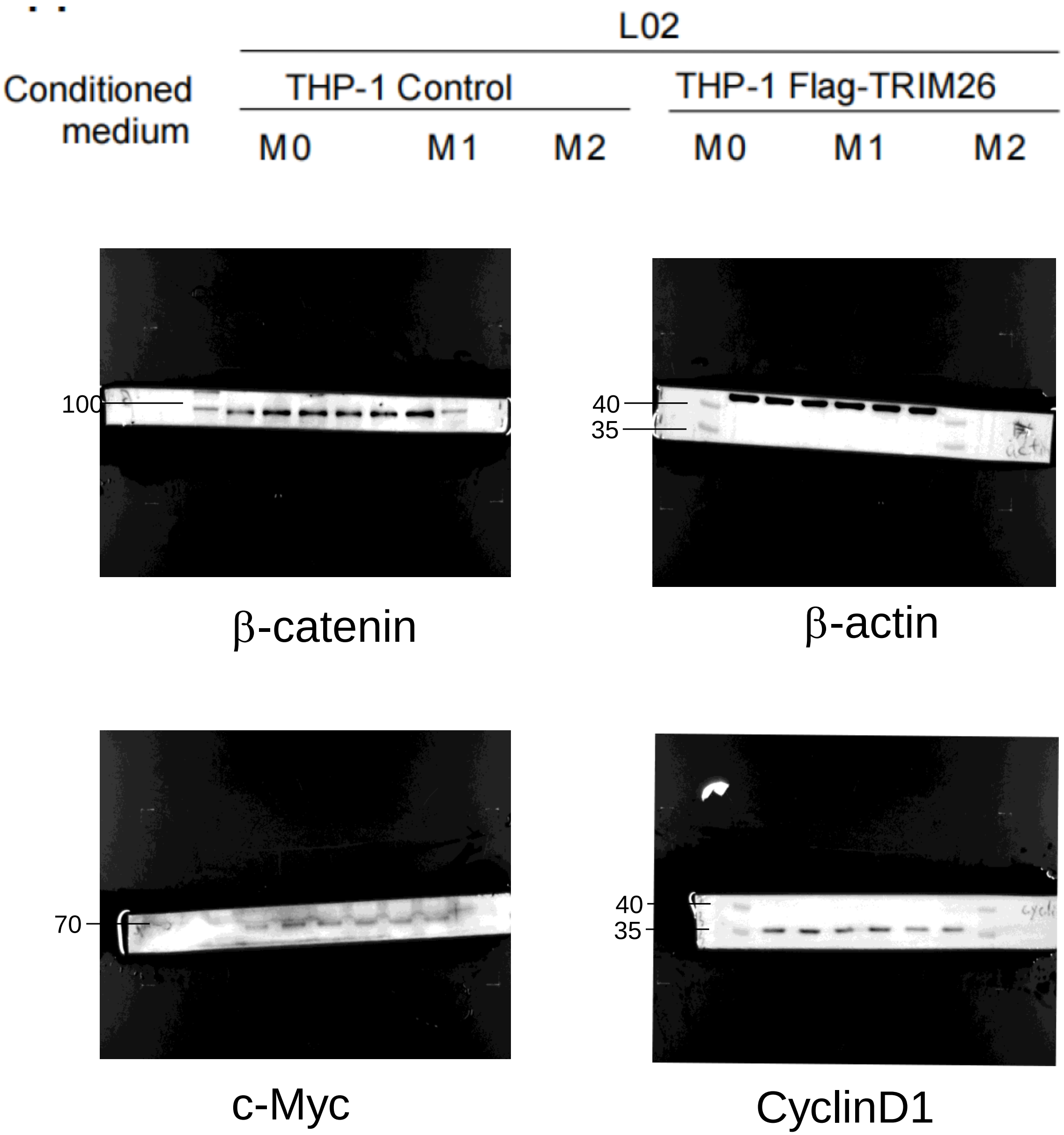

L02
Conditioned medium
THP-1 Control
THP-1 Flag-TRIM26
M0
M1
M2
M0
M1
M2
100
40
35
β-catenin
β-actin
70
40
35
c-Myc
CyclinD1

Fig 6I

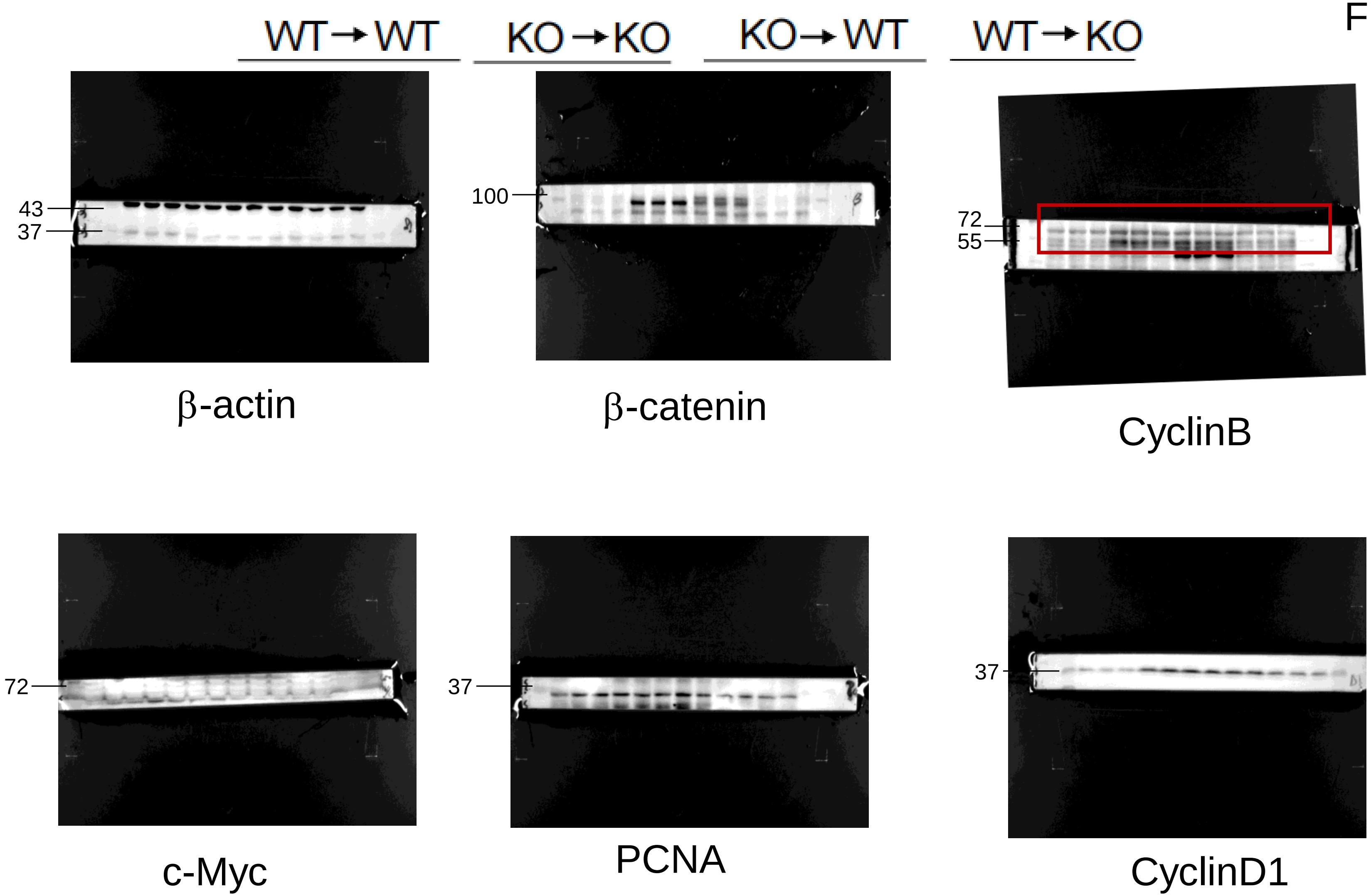

WT→WT
KO→KO
KO→WT
WT→KO
43
37
β-actin
100
β-catenin
72
55
CyclinB
72
c-Myc
37
PCNA
37
CyclinD1

Fig 7F

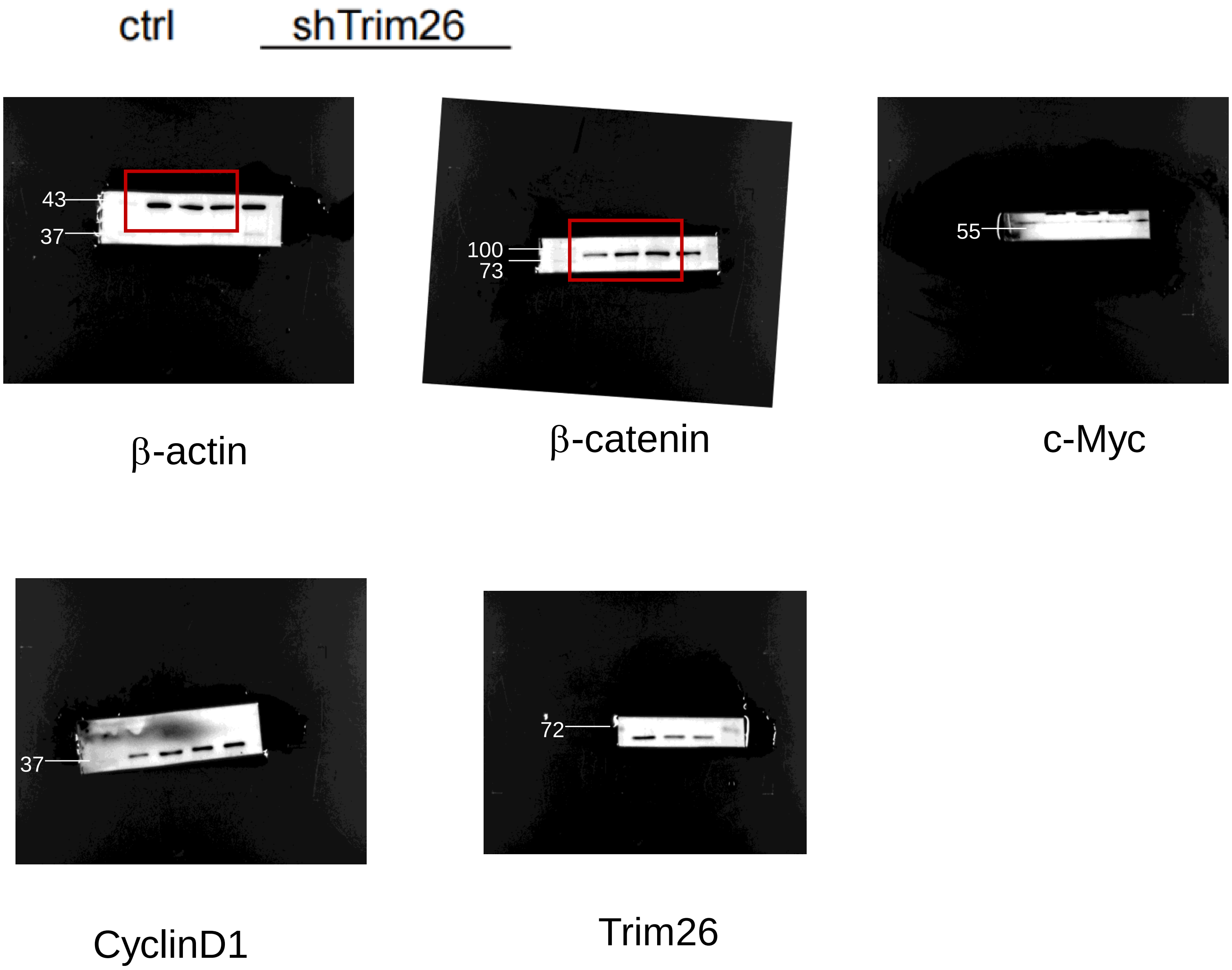

ctrl
shTrim26
43
37
100
73
55
β-actin
β-catenin
c-Myc
37
72
CyclinD1
Trim26

Fig 7G

Ctrl myc-*Trim26*

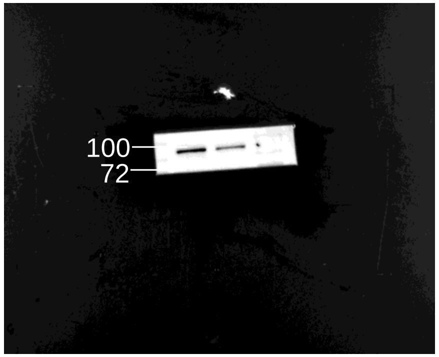

β-catenin

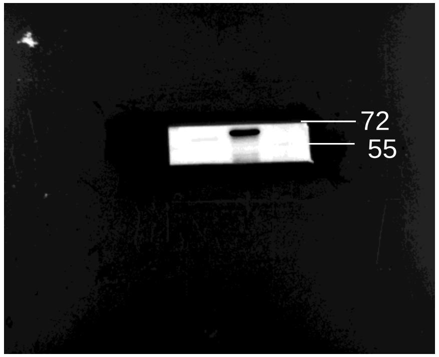

Trim26

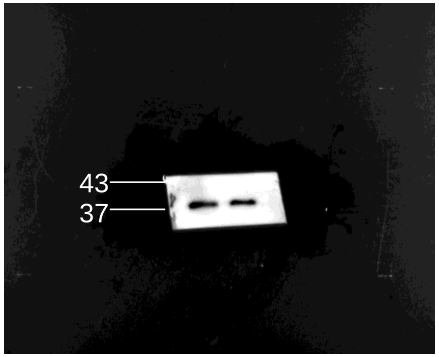

CyclinD1

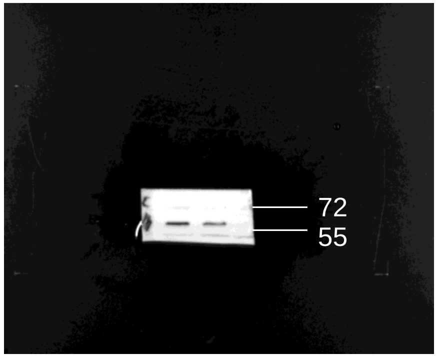

c-Myc

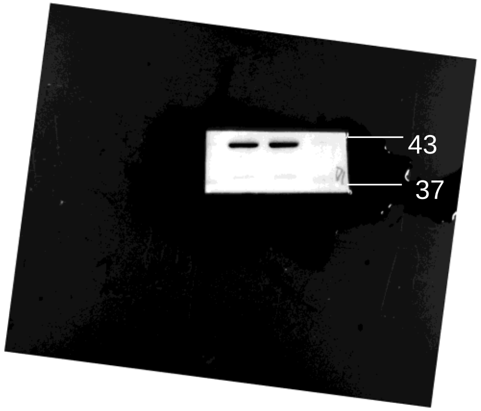

β-actin

Fig 7H

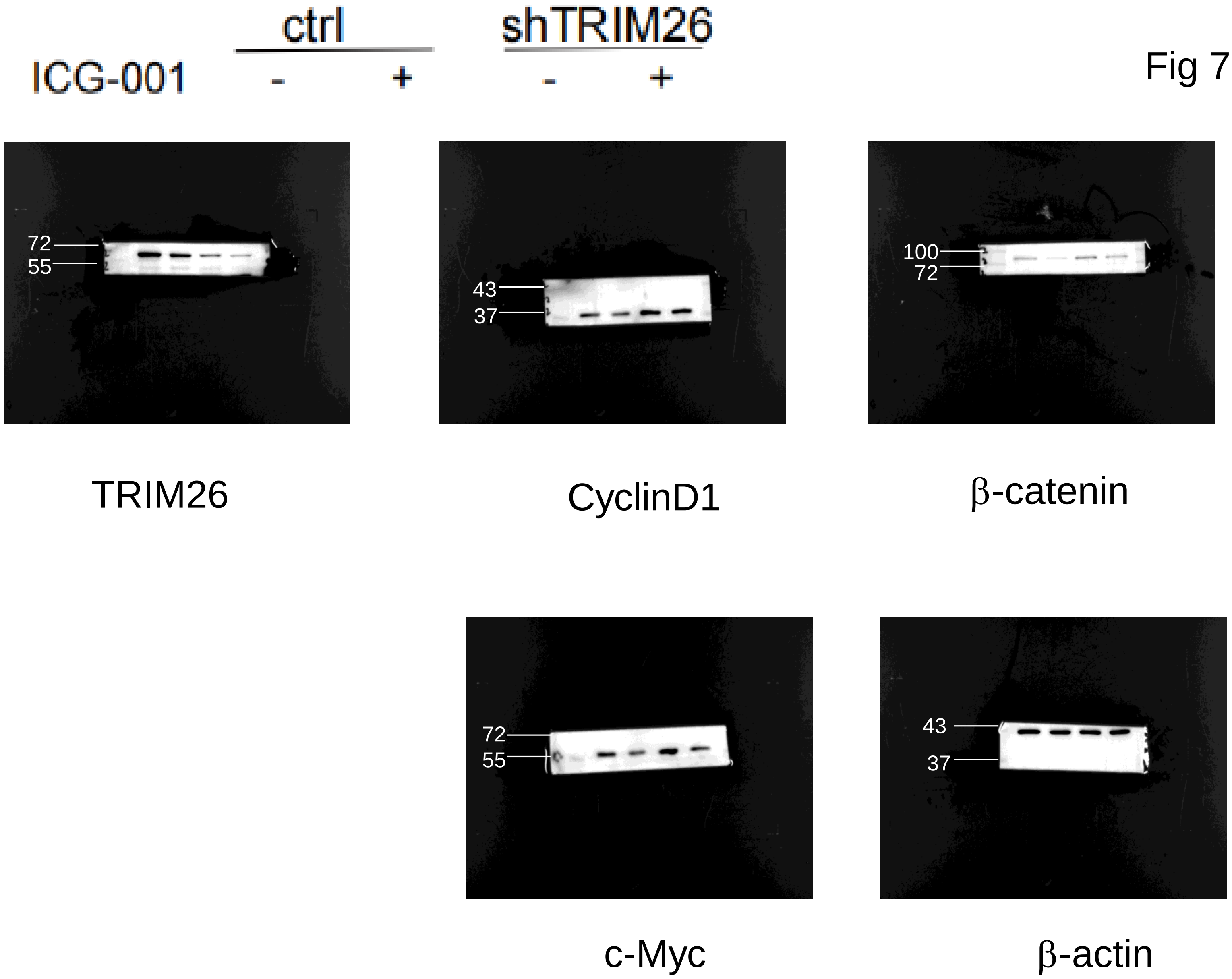
ctrl
shTRIM26
ICG-001
-
+
-
+
72
55
TRIM26
43
37
CyclinD1
100
72
β-catenin
72
55
c-Myc
43
37
β-actin

Fig 7I

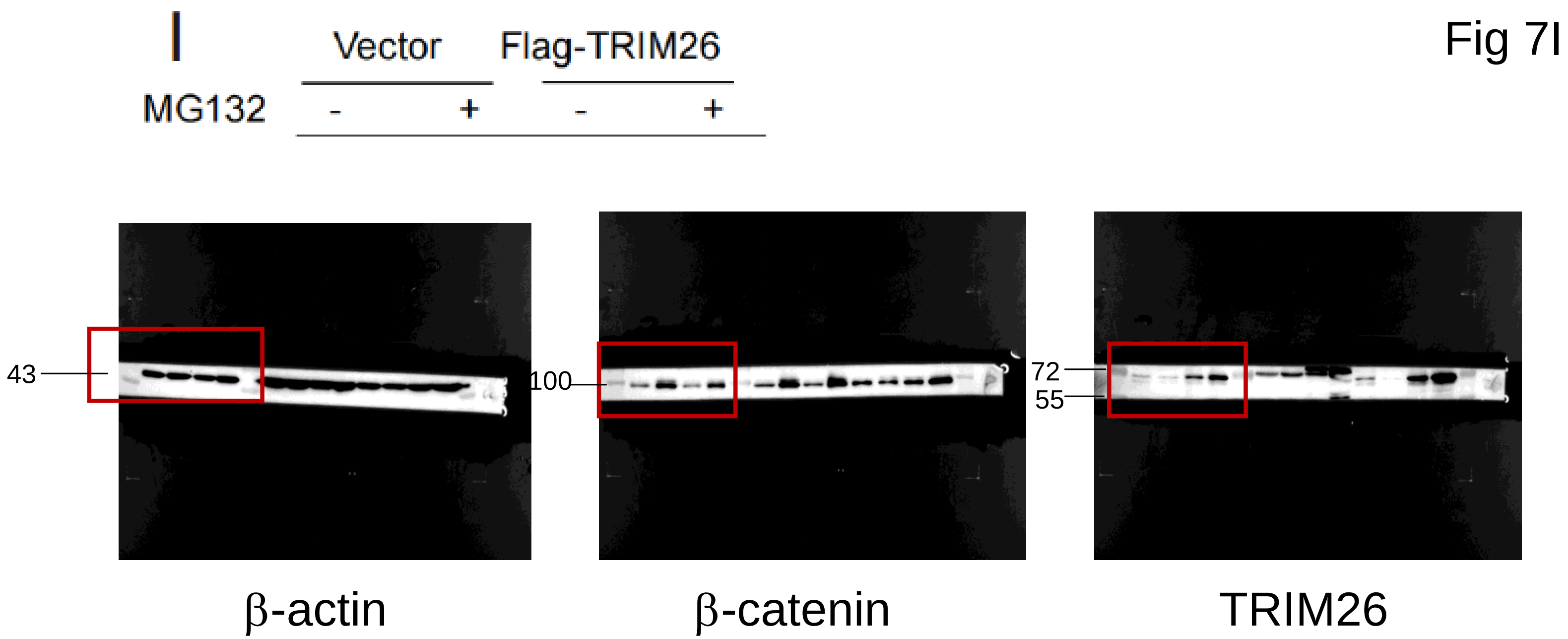
I
Vector
Flag-TRIM26
MG132
-
+
-
+
43
100
72
55
β-actin
β-catenin
TRIM26

Fig 7J

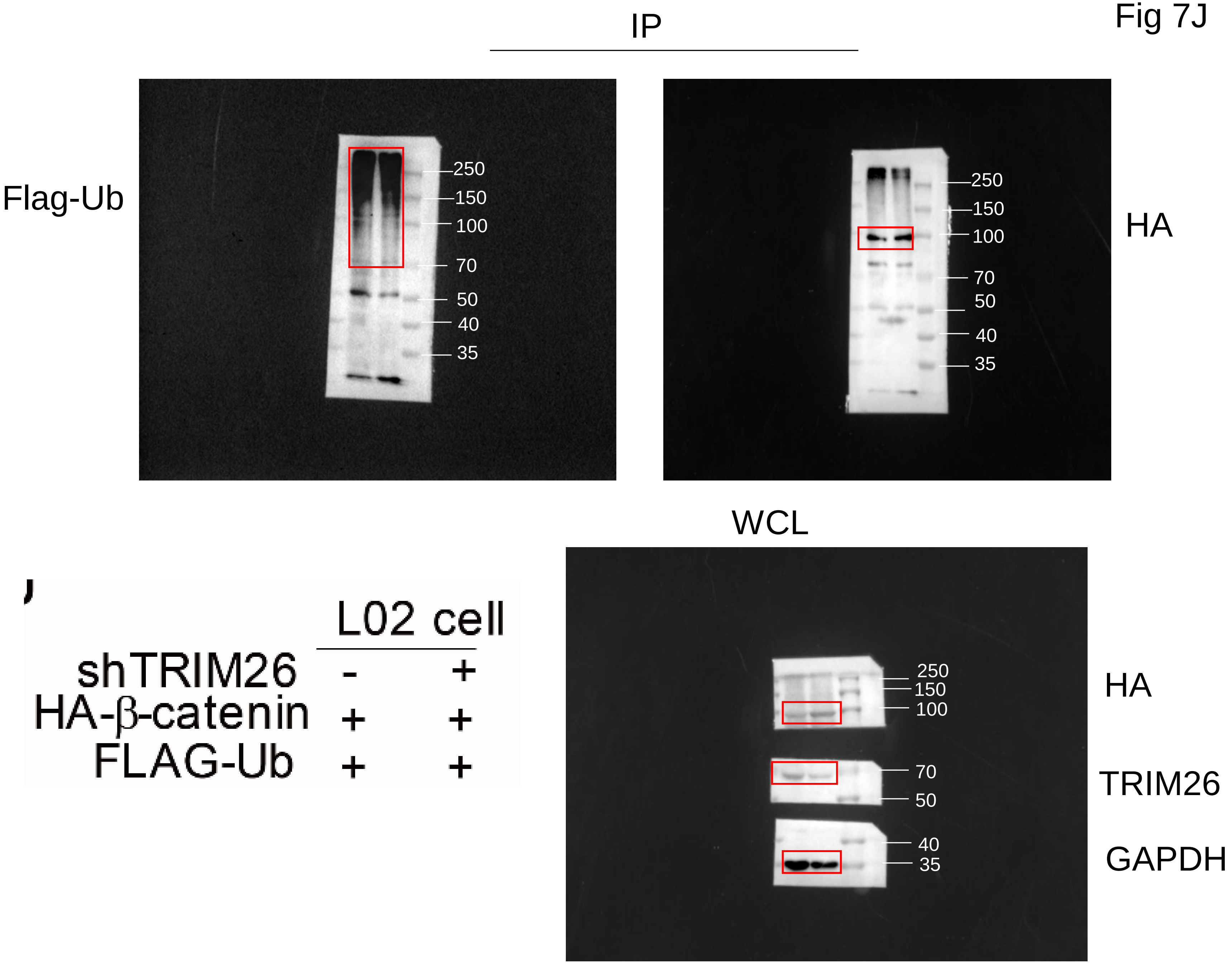
IP
Flag-Ub
250
150
100
70
50
40
35
HA
250
150
100
70
50
40
35
WCL
L02 cell
shTRIM26 - +
HA-β-catenin + +
FLAG-Ub + +
250
150
100
HA
70
50
TRIM26
40
35
GAPDH

Fig 8F

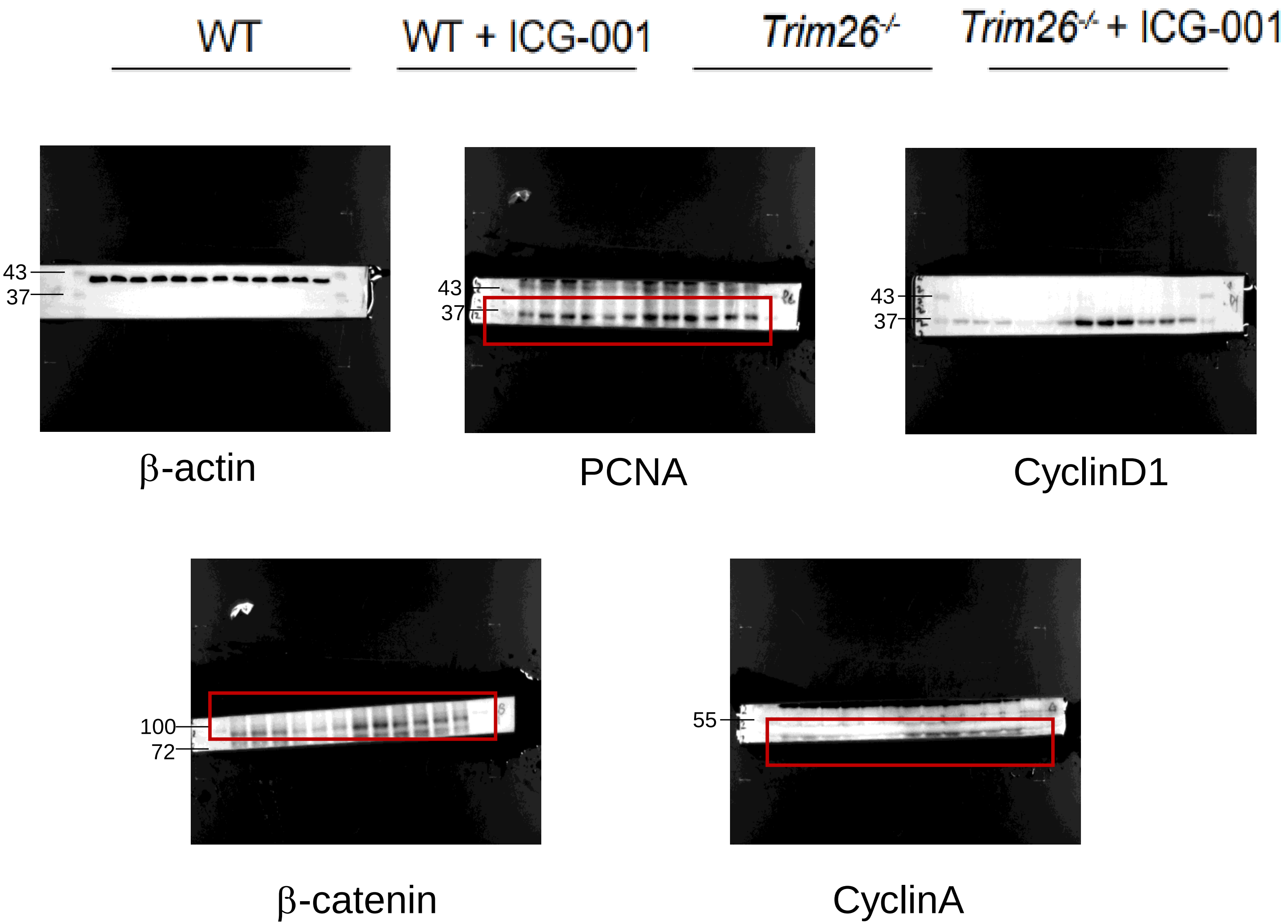

WT
WT + ICG-001
Trim26-/-
Trim26-/- + ICG-001
43
37
β-actin
43
37
PCNA
43
37
CyclinD1
100
72
β-catenin
55
CyclinA

Fig S1A

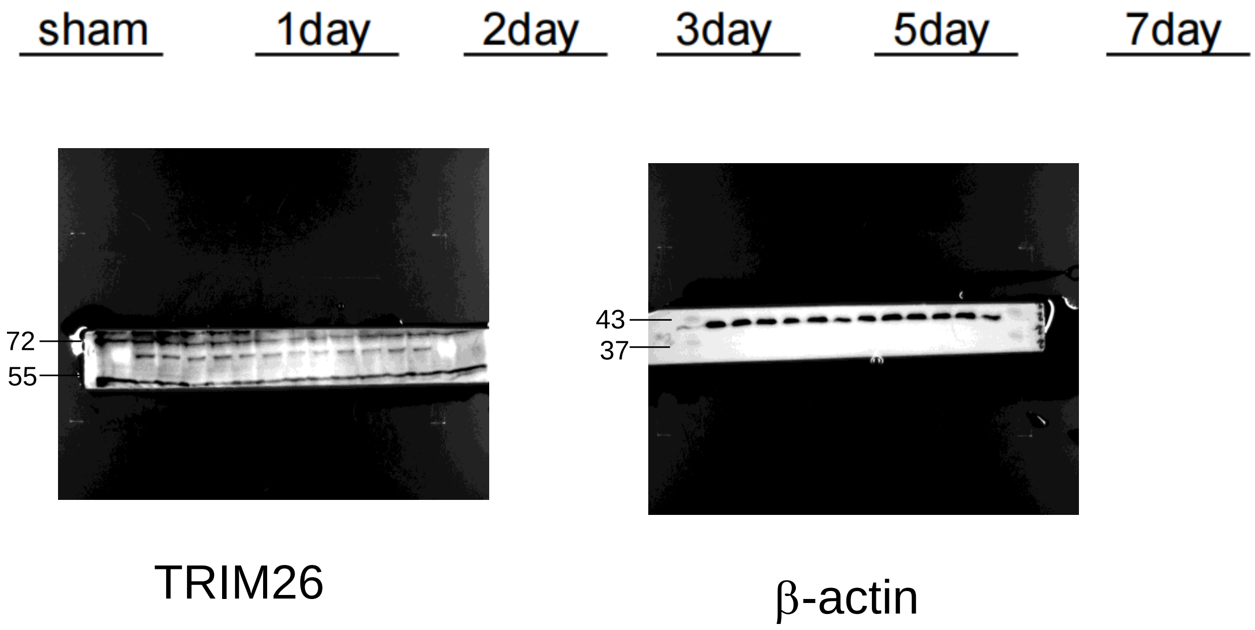

Fig S1B

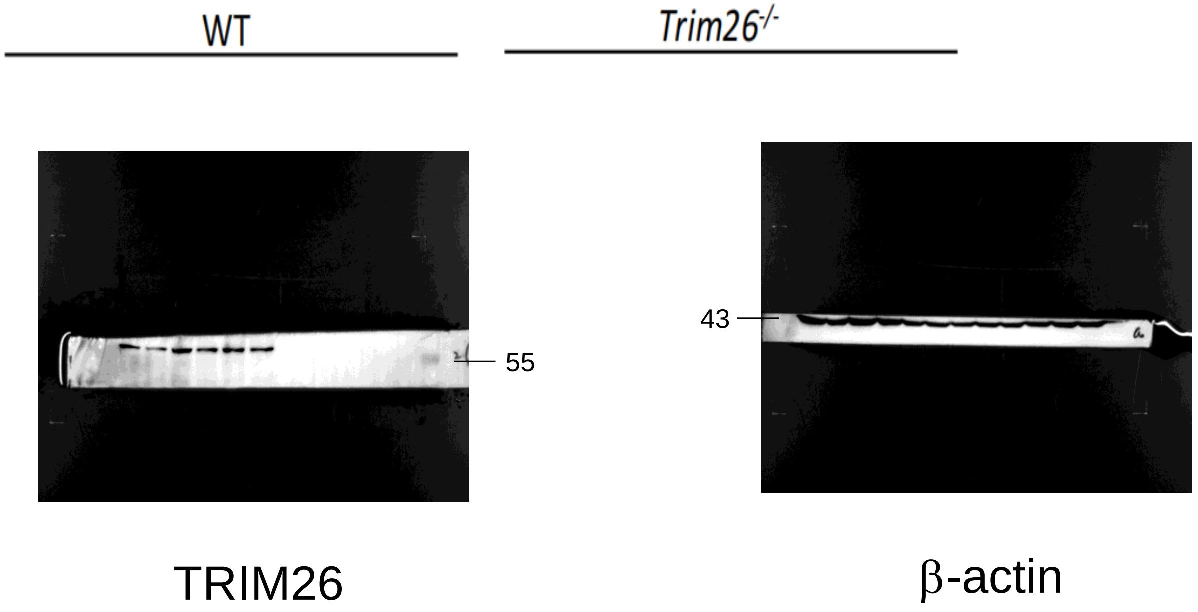

WT Ctrl Lip WT CLO Lip KO CLO Lip

Fig S3H

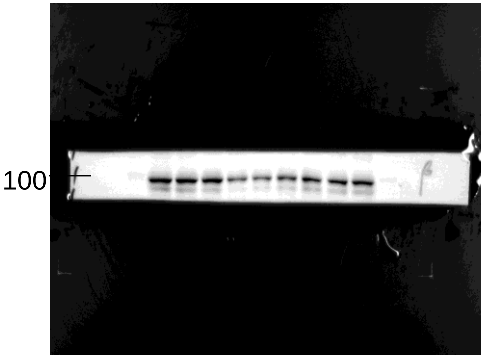

β-catenin

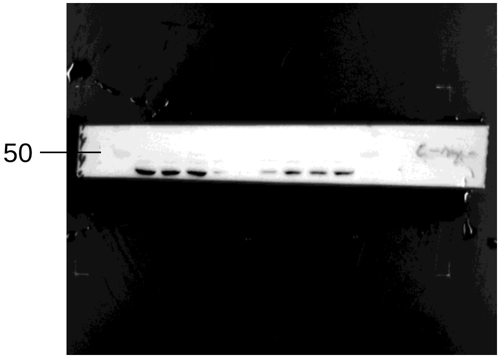

c-Myc

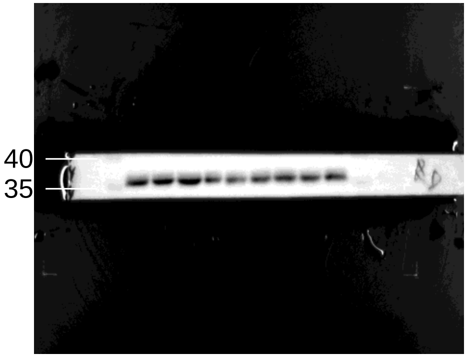

CyclinD1

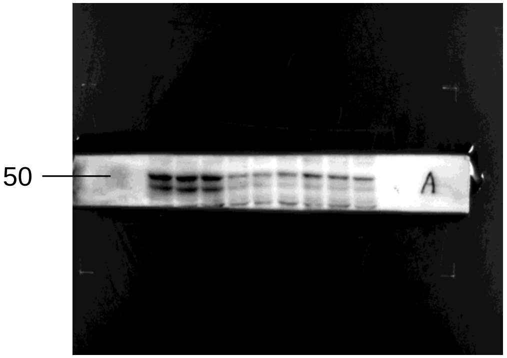

CyclinA2

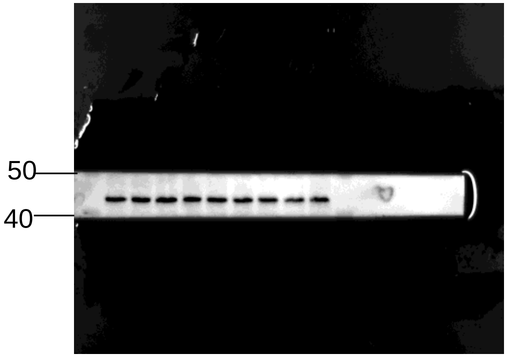

actin

Fig S6C

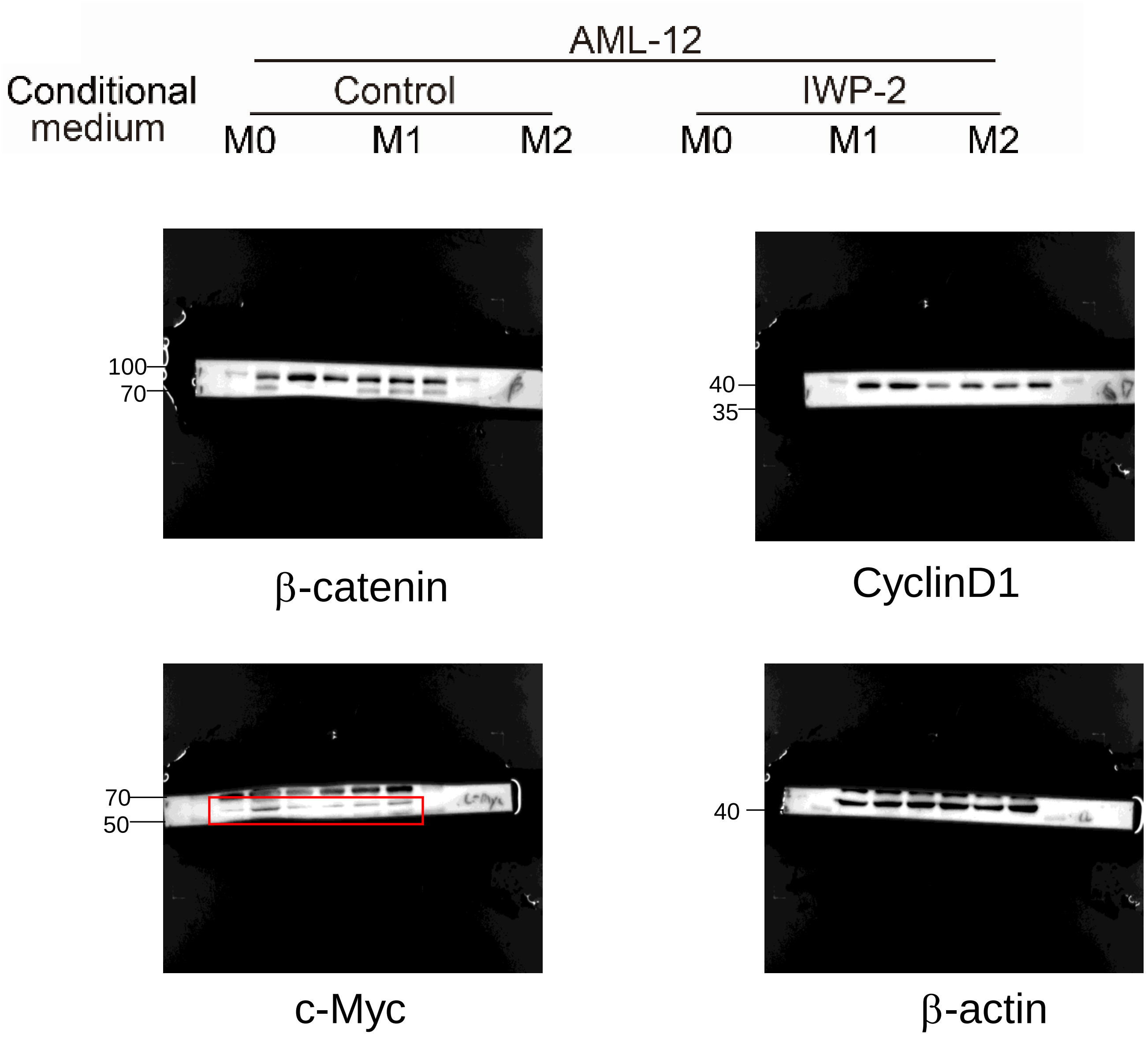
AML-12
Conditional medium
Control
IWP-2
M0
M1
M2
M0
M1
M2
100
70
β-catenin
40
35
CyclinD1
70
50
c-Myc
40
β-actin
